# Photothermal‐enhanced in situ supramolecular hydrogel promotes bacteria‐infected wound healing in diabetes

**DOI:** 10.1002/SMMD.20230047

**Published:** 2024-02-23

**Authors:** Chen Zheng, Xuan Wu, Ming Liu, Yulong Lan, Qian Liu, Erya Cai, Zhiyong Liao, Jianliang Shen

**Affiliations:** ^1^ College of Life and Environmental Science Wenzhou University Wenzhou Zhejiang China; ^2^ Zhejiang Engineering Research Center for Tissue Repair Materials Wenzhou Institute University of Chinese Academy of Sciences Wenzhou Zhejiang China; ^3^ National Engineering Research Center of Ophthalmology and Optometry Eye Hospital Wenzhou Medical University Wenzhou Zhejiang China; ^4^ School & Hospital of Stomatology Wenzhou Medical University Wenzhou Zhejiang China

**Keywords:** antibacterial, diabetic wound healing, *in situ* hydrogel, injectable hydrogel, photothermal therapy

## Abstract

Bacterial infection can impede the healing of chronic wounds, particularly diabetic wounds. The high‐sugar environment of diabetic wounds creates a favorable condition for bacterial growth, posing a challenge to wound healing. In clinical treatment, the irregular shape of the wound and the poor mechanical properties of traditional gel adjuvants make them susceptible to mechanical shear and compression, leading to morphological changes and fractures, and difficult to adapt to irregular wounds. Traditional gel adjuvants are prepared in advance, while in situ gel is formed at the site of administration after drug delivery in a liquid state, which can better fit the shape of the wound. Therefore, this study developed an in situ HA/GCA/Fe^2+^‐GOx gel using a photothermal‐enhanced Fenton reaction to promote the generation of hydroxyl radicals (·OH). The generation of ·OH has an antibacterial effect while promoting the formation of the gel, achieving a dual effect. The addition of double‐bonded adamantane (Ada) interacts with the host‐guest effect of graphene oxide and the double‐bond polymerization of HAMA gel, making the entire gel system more complete. At the same time, the storage modulus (G′) of the gel increased from 130 to 330 Pa, enhancing the mechanical properties of the gel. This enables the gel to have better injectability and self‐healing effects. The addition of GOx can consume glucose at the wound site, providing a good microenvironment for the repair of diabetic wounds. The gel has good biocompatibility and in a diabetic rat wound model infected with *S*. *aureus*, it can effectively kill bacteria at the wound site and promote wound repair. Meanwhile, the inflammation of wounds treated with HA/GCA/Fe^2+^‐GOx + NIR was lighter compared to untreated wounds. Therefore, this study provides a promising strategy for treating bacterial‐infected diabetic wounds.

## Graphical abstract

A light‐enhanced in situ gel‐forming hydrogel has been developed for treating bacterially infected diabetic wounds with the ability to have a dual effect by acting as an antimicrobial and forming a gel through hydroxyl radicals.

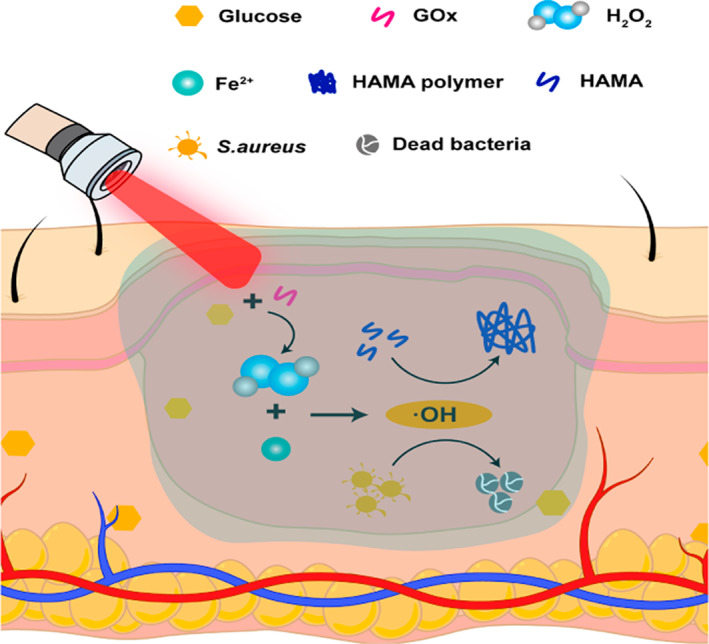

**Key points**

- *In situ* adhesive generation technology allows for more effective bonding of irregular wounds.
- Using glucose oxidase (GOx) to metabolize glucose thereby decreasing the sugar content in the wound area, while also supplying the raw materials for the Fenton reaction (H_2_O_2_).
- The hydroxyl radicals produced by the Fenton reaction serve a dual purpose: they aid in the gel formation and also act as antibacterial agents, achieving a two‐fold effect.
- The generated HA/GCA/Fe^2+^‐GOx gel structure was able to accelerate the wound healing process.

## 1 INTRODUCTION

The skin is an important organ that covers the surface of the body and is in direct contact with the external environment. As the body's first line of defense, it is also the most fragile and vulnerable organ.^1^ Although most injuries to the skin can be recovered within a certain time, secondary injuries, such as bacterial infection, during the repair process can prevent proper wound repair, especially in chronic wounds.^2^ Diabetic wounds, one of the chronic wounds, provide a favorable environment for bacterial growth due to the high glycemic microenvironment, leading to a high value‐added of pathogenic bacteria making diabetic wound repair impeded.^3^ Therefore, the healing of infected diabetic wounds has been a challenge. To realize the high efficiency of wound healing, tremendous wound dressings have been designed and fabricated to deliver specific therapeutic agents to the infected diabetic wounds, which could regulate the microenvironment of diabetic wounds, as well as kill the bacteria in the wounds.^4^ Among these wound dressings, hydrogels have attracted tremendous attention in the past decades, due to their three‐dimensional porous structures, which exhibit high drug delivery efficiency, provide a desired environment for cellular proliferation, and so on.^5^ However, in clinical treatments, the shapes of wounds are usually irregular. To realize the perfect coverage of these irregular wounds, some specific properties should be qualified in these designed hydrogels, especially the in situ formation ability.

The in situ formed hydrogels indicated the small molecules or macromolecules transferred into a 3D crosslinked network through covalent or non‐covalent interactions under external stimuli.^6^ *In situ*‐formed hydrogels provide better coverage of irregular, deep wounds than pre‐prepared gels.^7^ Common methods for constructing in situ hydrogels include physical cross‐linking and chemical cross‐linking.^8^ Compared with non‐covalent bond‐linked hydrogels, the covalent bond‐linked hydrogels exhibited excellent properties in stability, which would enable an excellent candidate for hydroxyl radical‐mediated polymerization and attracted tremendous attention, due to their excellent properties in antibacterial efficiency.^9^ Therefore, the *in situ*‐formed hydroxyl radical could present two major functions, one was killing the bacteria in the wound areas, and the other was acting as the trigger for the hydrogels.^10^

As is well known, the hydroxyl radicals (·OH) could be efficiently obtained through the Fenton reaction, in which the H_2_O_2_ could be converted to ·OH in the presence of Fe^2+^.^11^ Moreover, H_2_O_2_ could be found in the inflammatory microenvironment, especially in the diabetic wound. The high blood sugar microenvironment of a diabetic wound causes bacteria to grow faster than in a normal wound and also prevents the immune system from killing invading bacteria.^12^ A common method is to convert glucose to H_2_O_2_ via GOx and then consume the H_2_O_2_.^13^ Using the above strategy, glucose can be converted into ·OH, which in turn can be utilized antimicrobial and formed into a gel, which plays a crucial role in promoting the repair of bacterially infected diabetic wounds.

**Scheme 1:**
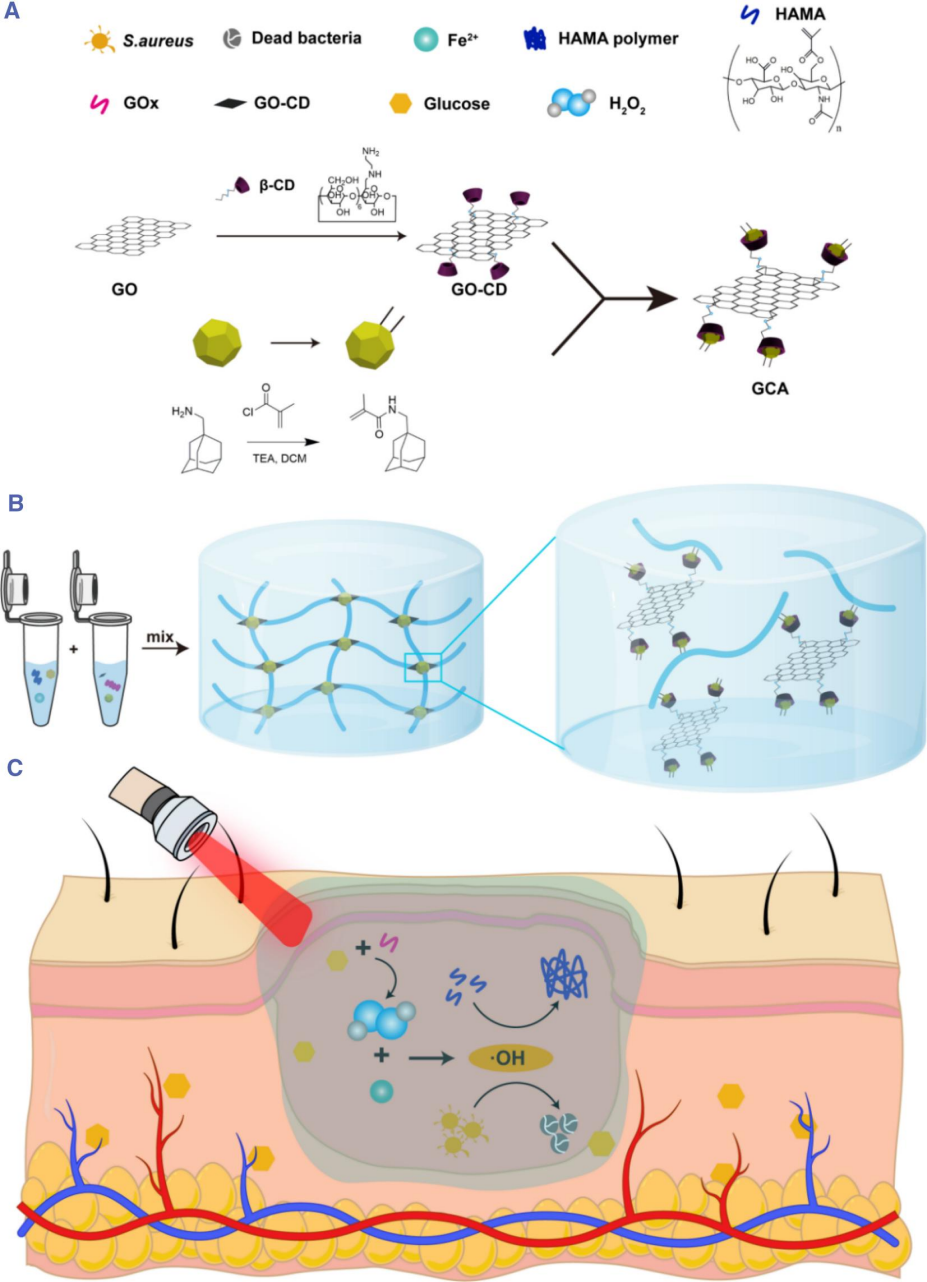
Schematic illustration of photothermal enhanced in situ supramolecular hydrogel for bacterially infected diabetic wounds. (A) Synthesis pathways for GCA materials. (B) Ada connects the gel system as a whole through the subject‐object interaction. (C) Diagram of the antimicrobial and gel‐forming mechanism of HA/GCA/Fe^2+^‐GOx gels. By heating with near‐infrared light, the Fenton reaction is promoted to generate ·OH, which is used to produce a gel with antimicrobial properties.

In this study, we prepared an in situ formed photothermal enhanced synergistic antimicrobial hydrogel (Scheme 1). Specifically, we prepared gels of HAMA, GOx, FeSO_4_, GO‐CD, and Ada using a one‐step process. In this case, GO‐CD and adamantane are interconnected through host‐guest interactions, whereas HAMA monomers can be polymerized by free radical polymerization to generate gels. In this system, glucose can be converted into H_2_O_2_, while Fe^2+^ consumes H_2_O_2_ to produce ·OH, which is produced partly for antibacterial purposes and partly for gel formation. The photothermal properties of GO‐CD were utilized to accelerate the Fenton reaction in a microthermal environment. Grafted double‐bonded adamantane, on the other hand, is better able to link the GO‐CD to the HAMA gel system, making it a tighter whole. Finally, the HA/GCA/Fe^2+^‐GOx gel system was verified by in vivo experiments to have a telling effect on the repair of bacterially infected diabetic wounds.

## 2 RESULTS

### 2.1 Characterization of GO‐CD and Ada

**FIGURE 1:**
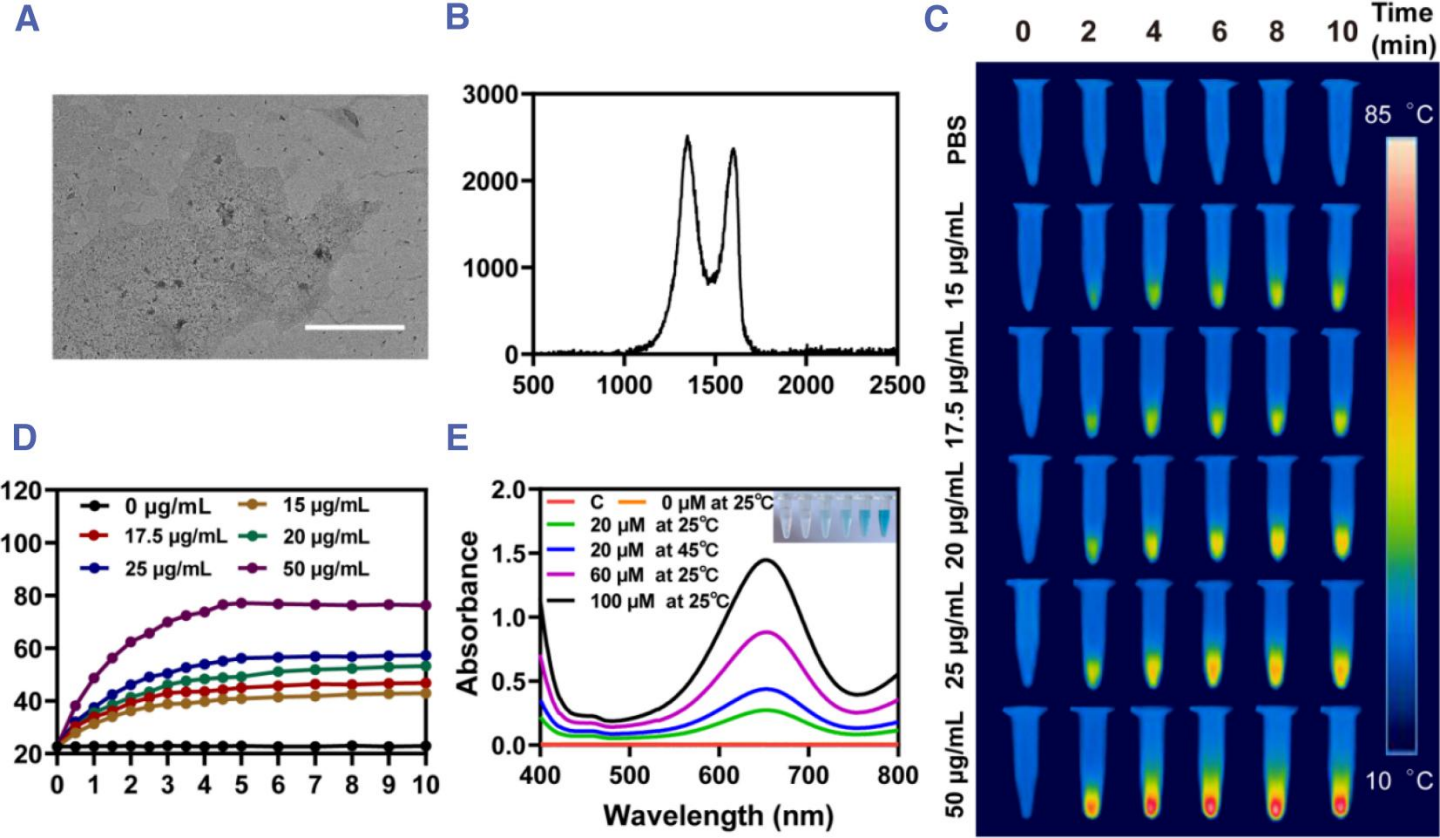
Preparation and characterization of GO‐CD. (A) TEM image of GO‐CD. (B) Raman spectrogram of GO‐CD (scale bar = 500 μm). (C) Thermograms of PBS versus GO‐CD at different concentrations (15, 17.5, 20, 25, and 50 μg/mL). (D) Warming curves of PBS with different concentrations of GO‐CD (15, 17.5, 20, 25 and 50 μg/mL). (E) TMB detects ·OH produced by the Fenton reaction of different concentrations of FeSO_4_ with 100 μM H_2_O_2_ at different temperatures.

Graphene oxide (GO) is an oxide of graphene which is oxidized to increase the number of oxygen‐containing functional groups and become more reactive than graphene.^14^ GO's unique mechanical, electronic, and optical properties make it useful in biotechnology,^15^ biomedical engineering,^16^ and tissue engineering.^17^ Therefore, we prepared β‐CD functionalized graphene nanosheets. With the TEM image, it can be observed that GO‐CD possesses a lamellar structure like GO (Figure 1A). Then, the Raman spectrum shows that there is a characteristic peak called D peak at around 1350 cm^−1^ and a characteristic peak called G peak at around 1580 cm^−1^ (Figure 1B). The D peak is due to the change of the benzene‐like ring from a carbon‐carbon double bond to a carbon‐carbon single bond on the oxidized graphite in graphene oxide, forming sp^3^ hybridized carbon atoms, and sp^2^ hybridized carbon atoms correspond to the G peak.

As seen by the results of FTIR spectra (Figure S1), the GO characteristic peaks due to GO carboxylate (COOH) and carboxylate (COO‐) vibrations are at 1721 and 1110 cm^−1^, respectively. While β‐CD has a sugar ring twisting vibration peak at 600–800 cm^−1^. 3400–3600 cm^−1^ is caused by hydroxyl (OH) vibrations present in both GO and β‐CD. The peak around 2800 cm^−1^ is due to the covalent interaction of GO with β‐CD C‐H. The results demonstrated that β‐CD was successfully grafted to GO. β‐CD is a novel molecular encapsulation material which can encapsulate adamantane through host‐guest interaction. To make the system more compact and complete, we prepared adamantane with a grafted double bond (Ada), and the NMR results showed that the double bond was successfully accessed in adamantane (Figure S2). The addition of adamantane enables the gel to be better connected to the material as a complete system. We will refer to the system of GO‐CD wrapped Ada as GCA for short.

At the same time, graphene has a good effect on light and heat conversion. Therefore, the synthesized GO‐CD was screened for photothermal concentration (Figure 1C). Using phosphate‐buffered saline (PBS) as a control group, it can be seen that PBS has almost no temperature change under 808 NIR 1 W/cm^2^ irradiation. While different concentrations of GO‐CD (15, 17.5, 20, 25, 50 μg/mL) almost reached the highest temperature after 5 min of light irradiation and remained constant. Their maximum temperatures were around 41°C, 45°C, 51°C, 56°C, and 76°C, respectively (Figure 1D). Finally, we chose the concentration of GO‐CD = 17.5 μg/mL, at which its highest temperature can reach 45°C.

### 2.2 Detection of hydroxyl radicals

In recent years, many effective methods have been utilized in the treatment of antimicrobials, such as chemotherapy,^18^ photodynamic^19^ and sonodynamic therapy.^20^ The Fenton reaction is characterized by simplicity of operation, rapidity of reaction, and low cost of use. The Fenton reaction is capable of producing ·OH, which can cause irreversible damage to the cell walls of bacteria.^21^ To demonstrate more visually that the Fenton reaction can produce ·OH. We utilized TMB to detect the generation of free radicals.

We fixed the amount of H_2_O_2_ to 100 μM and then mixed it with different concentrations of FeSO_4_ (20, 60, and 100 μM) for the reaction (Figure 1E). And ·OH production was detected at room temperature as well as under microthermal conditions. From the results, it can be seen that 100 μM of H_2_O_2_ by itself does not cause discoloration of the TMB, and only if divalent iron and H_2_O_2_ are present at the same time, their reaction can produce ·OH. The higher Fe^2+^ content produces more ·OH, resulting in a darker TMB color and its absorption peak at 625 nm is higher. We selected 20 μM FeSO_4_ for the comparison of ·OH production under room temperature and microthermal conditions. It was found that the reaction between the same concentration of H_2_O_2_ and FeSO_4_ was able to produce a more ·OH under slightly hot (45°C) conditions. Meanwhile, we kept the Fe^2+^ concentration constant (100 μM) and tested different concentrations of hydrogen peroxide at the same reaction time to determine the concentration of hydrogen peroxide catalyzed by the enzyme by assaying and evaluating the color development of TMB (Figure S3).

To further detect ·OH, we also used TA for ·OH detection (Figure S4). As shown in the experimental results, TA, TA + H_2_O_2_ (100 μM) and TA + FeSO_4_ (20 μM) do not fluoresce. Only H_2_O_2_ (100 μM) and FeSO_4_ (20 μM) are present at the same time, and H_2_O_2_ reacts with Fe^2+^ in a Fenton reaction, and TA reacts with ·OH to produce fluorescence. It can also be seen that the fluorescence intensity at 45°C is greater than that at 25°C. This also indicates that the production of hydroxyl radicals can be enhanced under warm conditions (45°C).

To demonstrate that under microthermal conditions (45°C) can promote the onset of Fenton action to enhance gel generation, we chose HAMA (2%, w/v), FeSO_4_ (2 mM), GO‐CD (17.5 μg/mL), GOx (10 μg/mL) and Ada (8 μg/mL) for group experiments. We divided into a without NIR group and a with NIR group to compare the time of gel formation. As Figure S5 shows, in the absence of 808 NIR 1 W/cm^2^ presence, it took about 10 min to generate a gel, whereas in the presence of 808 NIR 1 W/cm^2^ illumination, the gel time was reduced to 5 min. This suggests that microthermal conditions can accelerate the production of ·OH, thereby promoting gel formation.

### 2.3 Preparation and characterization of hydrogels

**FIGURE 2:**
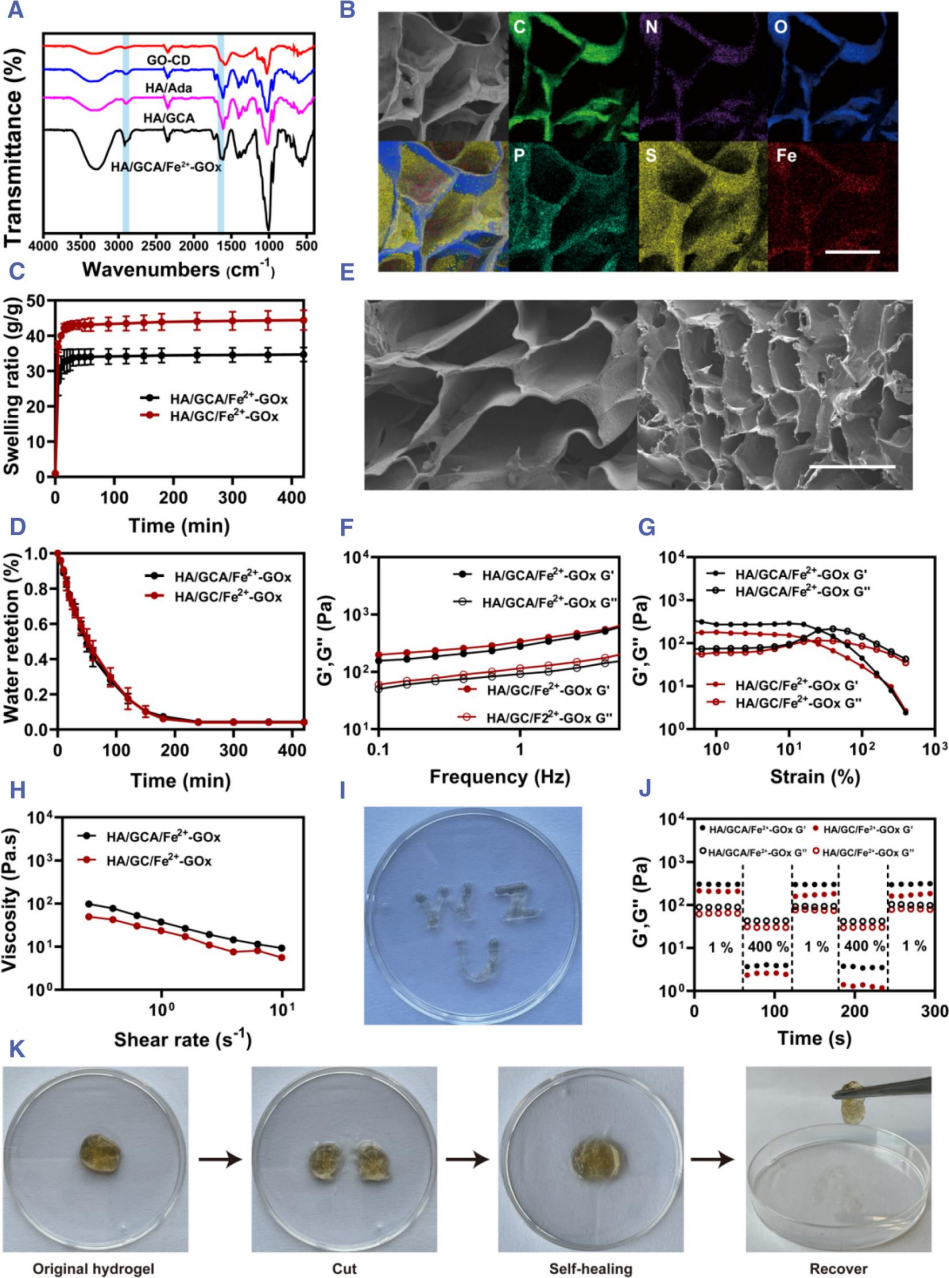
Characterization of hydrogel materials. (A) FTIR spectra of GO‐CD, HA/Ada, HA/GCA, and HA/GCA/Fe^2+^‐GOx. (B) Polymer elemental analysis (scale bar = 100 μm). (C) Dissolution rates of HA/GC/Fe^2+^‐GOx and HA/GCA/Fe^2+^‐GOx (*n* = 3). (D) Water retention of HA/GC/Fe^2+^‐GOx and HA/GCA/Fe^2+^‐GOx (*n* = 3). (E) SEM image of gel pore size HA/GC/Fe^2+^‐GOx (left) and HA/GCA/Fe^2+^‐GOx (right) (scale bar = 200 μm). (F) Frequency‐dependent rheological behavior of HA/GC/Fe^2+^‐GOx and HA/GCA/Fe^2+^‐GOx (frequency = 0.1 Hz). (G) HA/GC/Fe^2+^‐GOx and HA/GCA/Fe^2+^‐GOx's strain‐dependent rheological behavior (strain = 1%). (H) Shear denaturation behavior of HA/GCA/Fe^2+^‐GOx. (I) Injectability of HA/GCA/Fe^2+^‐GOx. (J) Self‐healing properties of HA/GCA/Fe^2+^‐GOx. (K) HA/GCA/Fe^2+^‐GOx self‐healing behavior.

Firstly, the chemical structure of HA/GCA/Fe^2+^‐GOx gels were investigated using FTIR. Among them, the small peak change around 1700 cm^−1^ is caused by the interaction of carboxyl groups with Fe, and the peak shape change around 2800 cm^−1^ is due to the encapsulation of adamantane by β‐cyclodextrins (Figure 2A). At the same time, the gels were analyzed for energetic elements. It is also evident from the energy spectrum that the gel contains C, N, O, P, S, Fe, and other ions all uniformly distributed on the gel surface (Figure 2B). Where the presence of the elements P and S is due to the presence of GOx. At the same time, we examined the ability of GOx to consume glucose as well as to produce H_2_O_2_ (Figure S6). We prepared a PBS solution with a glucose concentration of 11.1 mol/L and treated it with 10 μg/mL of GOx in 5 min. From the experimental results, it can be seen that the concentration of glucose decreased after GOx treatment, while the concentration of H_2_O_2_ increased.

To demonstrate that the addition of adamantane can make the gel system more compact, we prepared two gels, HA/GC/Fe^2+^‐GOx and HA/GCA/Fe^2+^‐GOx. We looked at the gels' swelling rate because, in the extremely porous environment of hydrogels, their capacity to absorb and hold water is a crucial factor in choosing an appropriate dressing. A comparison of the dissolution rates of the two gels, HA/GC/Fe^2+^‐GOx and HA/GCA/Fe^2+^‐GOx, can be visualized more visually through the graphs (Figure 2C). In the first 5 min, both gels absorb moisture quickly. In the following 240 min, the water uptake of the gel starts to become progressively slower until it finally reaches equilibrium. The dissolution rates of HA/GC/Fe^2+^‐GOx and HA/GCA/Fe^2+^‐GOx finally reached 45 and 34, respectively. In addition, the water retention properties of the hydrogels were investigated. In addition, the water retention properties of the hydrogels were investigated (Figure 2D). The water loss of HA/GCA/Fe^2+^‐GOx is somewhat less than that of HA/GC/Fe^2+^‐GOx. This difference in water loss could be ascribed to the addition of Ada, which improves the gel's cross‐linking degree and makes it more compact.

Subsequently, the internal micromorphology and skeletal structure of the lyophilized hydrogels were investigated by scanning electron microscopy. It is worth noting that the presence of Ada makes the gel system more complete and compact, so it can be seen that the HA/GCA/Fe^2+^‐GOx gel has smaller pores (100 μm) compared to the HA/GC/Fe^2+^‐GOx gel (200 μm) (Figure 2E). It was able to demonstrate that the results of gel swelling were related to the pore size of the gel. It was also demonstrated that the addition of Ada resulted in a tighter cross‐linking of the gel and a smaller pore size.

### 2.4 Mechanical studies of hydrogels

Since the addition of Ada induces changes in the microstructure of the gels that may affect the mechanical properties of the gels, the rheological parameters associated with the two gels with and without the addition of Ada were examined. A DHR‐2 rheometer with 1 and 25 mm plate geometries was used to extensively examine the rheological characteristics of injectable HA/GCA/Fe^2+^‐GOx hydrogels. First, we performed strain and frequency scans on the gels to better understand the hydrogel regime (Figure 2F). The energy storage modulus (G′) is consistently larger than the loss modulus (G″) in the absence of shearing of the sample. All tend to be more stable, which is characteristic of the formation of a stable gel. In particular, the addition of Ada increased the G′ of the hydrogel from 130 ± 10 Pa to 330 ± 10 Pa. This is due to the ability of Ada to be grafted in the cavities of cyclodextrins through the host‐guest reaction, and the double bond attached to Ada prompts the polymerization of the double bond with the HAMA gel, which results in a more compact structure of the gel. Nevertheless, G′ consistently falls and G″ progressively rises when the instrument starts to shear (Figure 2G). G″ starts to progressively surpass G′ at the yield point (strain ≈25%), at which time the sample transitions into a viscoelastic liquid.

Next, we determined the viscosity of the hydrogel (Figure 2H). Because of the network's dynamic physical cross‐linking locations, the hydrogels underwent shear fracture as predicted, and their viscosity dropped as the shear rate rose. Using a 1 mL syringe, we repeatedly injected the HA/GCA/Fe^2+^‐GOx gel into the petri dish to further illustrate the hydrogel's injectability. As a result, the “WZU” font formed and remained in the hydrogel state (Figure 2I). The HA/GCA/Fe^2+^‐GOx hydrogel's recoverability was then assessed using three continuous cyclic strain experiments. As the strain increased to 400%, Figure 2J shows how the hydrogel overshot the critical strain and changed into a sol state. But the HA/GCA/Fe^2+^‐GOx hydrogel reassembled and the moduli (G′ and G″) went back to their starting points when the strain dropped to 1%. It is noteworthy that even after two cycles, the hydrogel system exhibits a rapid sol‐gel transition characteristic. It suggests that the hydrogel system is capable of self‐healing. In the meantime, the intact gel underwent self‐healing for five to 10 min after being sliced (Figure 2K). These findings show that the hydrogel may cover and mend wounds because of its superior injectability and capacity for self‐healing. Simultaneously, we positioned the HA/GCA/Fe^2+^‐GOx gel preparations inside various model shapes and watched the gel form. Figure S7 illustrates how HA/GCA/Fe^2+^‐GOx gels may be formed into a multitude of shapes, suggesting that in situ gel creation can be used to modify the shape of the gels.

### 2.5 In vitro biocompatibility of hydrogels

**FIGURE 3:**
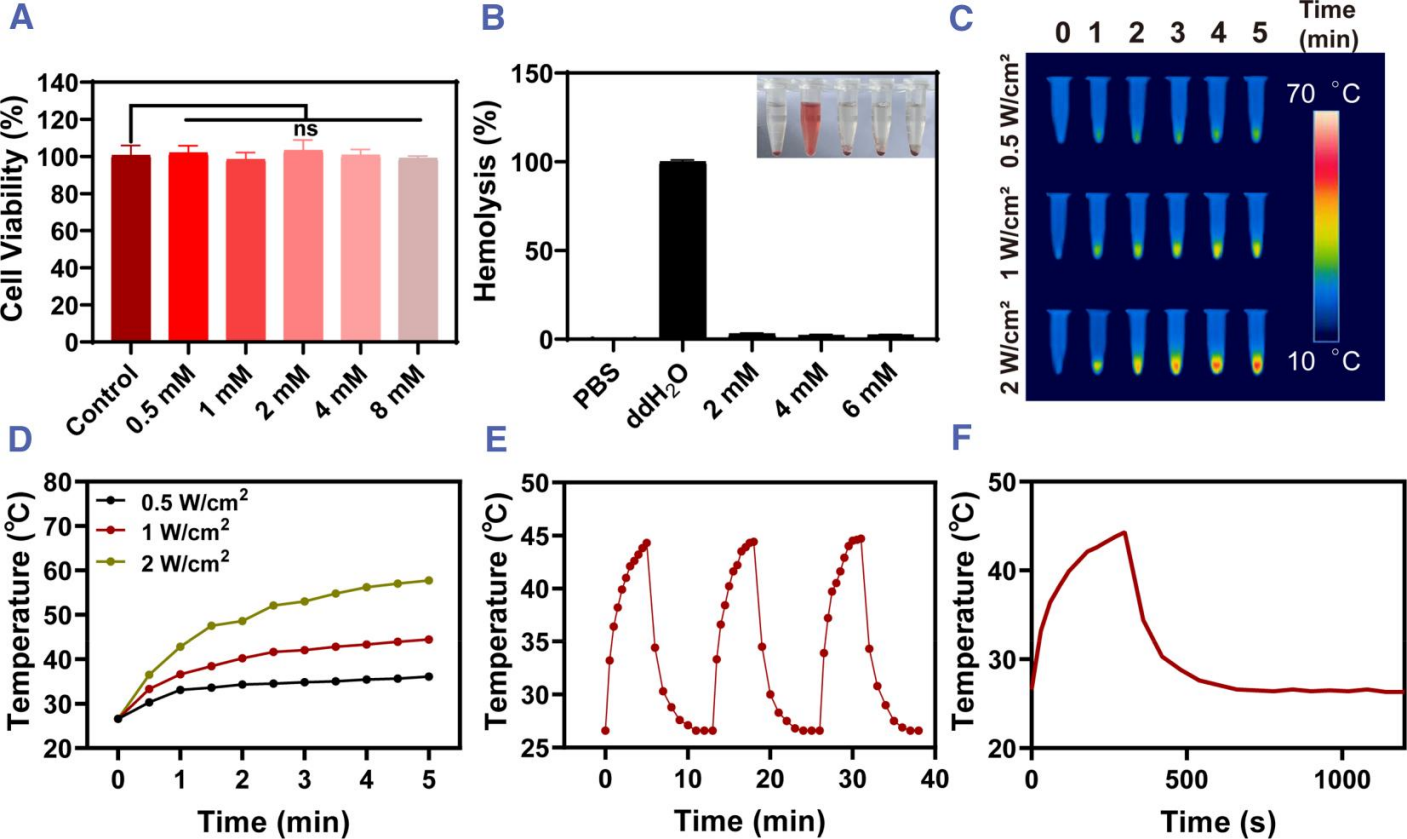
Biocompatibility and photothermal properties of HA/GCA/Fe^2+^‐GOx gel. (A) Cell viability of RS1 cells after 24 h incubation of gels leachates prepared with different concentrations of FeSO_4_ (*n* = 5). (B) Hemocompatibility testing of gels prepared with different concentrations of FeSO_4_ (*n* = 3). (C) Thermograms of gels irradiated by NIR lasers of different powers. (D) Warming curves of gels irradiated by NIR lasers of different powers. (E) Detection of photothermal stability properties of gels. (F) The photothermal conversion efficiency of the material was ascertained by examining the rise and fall diagram of the HA/GCA/Fe^2+^‐GOx gel's photothermal temperature. (NS means not significant).

Biocompatibility is essential for the biomedical use of hydrogel materials.^19^ RS1 and L929 cells were used to test the hydrogels' cytotoxicity in the beginning. After a day, the CCK‐8 assay showed that the cell viability of L929 cells (Figure S8) and RS1 cells (Figure 3A) was greater than 95%. The lack of a significant statistical difference between the hydrogel group and the control group (cells in culture media without hydrogel) revealed the hydrogel's good cell compatibility. Since the material would come into touch with blood from the wound site, we next looked at its blood compatibility (Figure 3B). Every hydrogel sample had a hemolysis rate of less than 5%, which is considered to be appropriate for hemostatic materials, and the supernatant is just as transparent as the negative control. These results imply that the hydrogel is biocompatible and appropriate for use as a dressing for tissue‐engineered wounds.

### 2.6 Photothermal antibacterial properties of hydrogels

Furthermore, the produced hydrogels' photothermal sterilization properties were examined. First, a thorough investigation into hydrogels' photothermal capacity was conducted. An 808 nm laser was utilized to irradiate the hydrogel; a control hydrogel without GO‐CD was also used. After screening, we determined that the final GO‐CD concentration of the gel system was 17.5 μg/mL. The gel photothermolysis was then screened with 0.5, 1, and 2 W/cm^2^ lasers, respectively (Figure 3C). It was found that their maximum temperatures reached 36°C, 45°C and 58°C, respectively (Figure 3D). The temperature raised by the final result 1 W/cm^2^ laser is optimal. It suggests that the photothermal of hydrogels is closely related not only to the concentration of the material but also to the power of the laser. Repeated heating and cooling processes had no significant effect on the photothermal effect of the gel, indicating that the material has stable photothermal stability (Figure 3E). The photothermal conversion rate is the key parameter of the photothermal material, which is calculated to be 45% (Figure 3F).^22^

**FIGURE 4:**
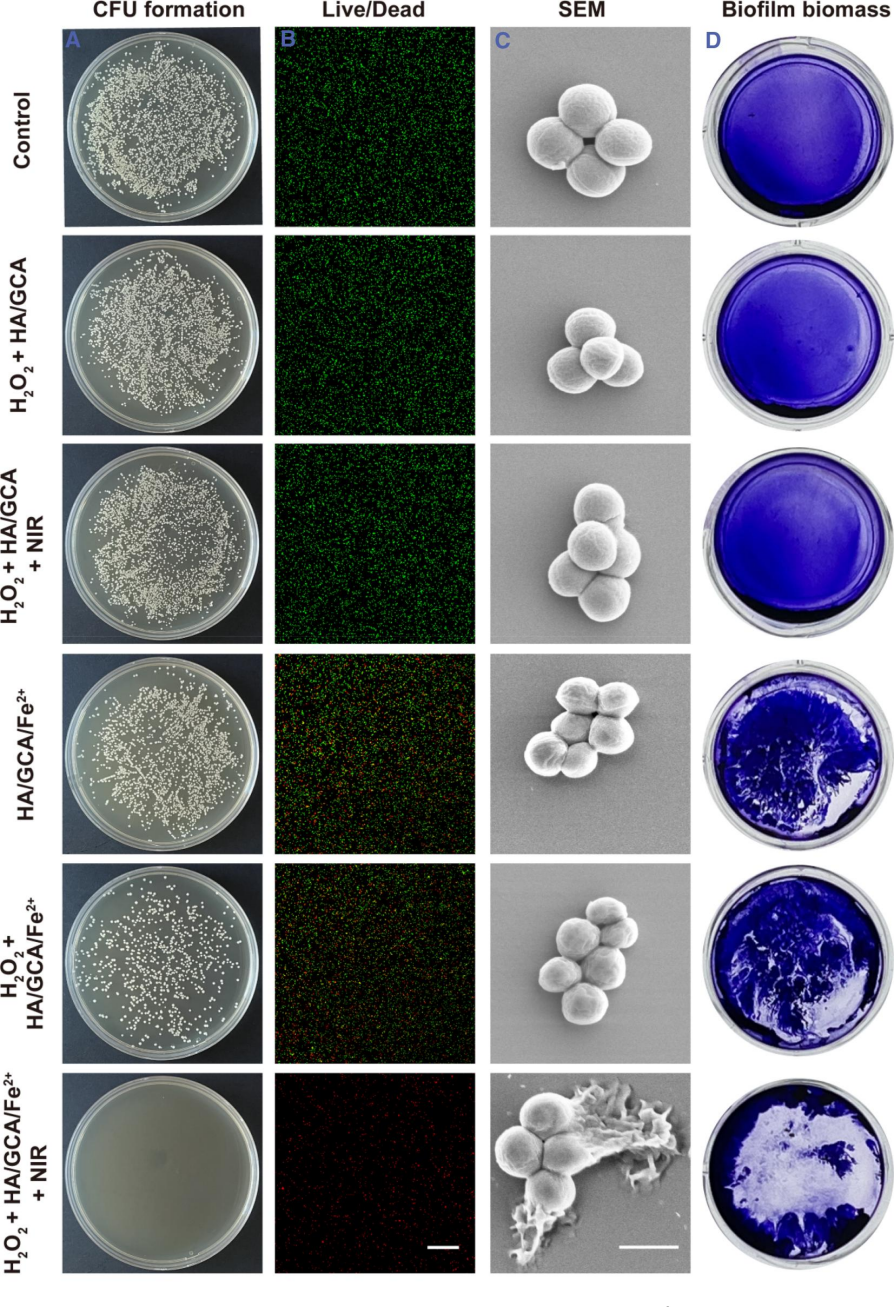
Photothermal antibacterial abilities of HA/GCA/Fe^2+^‐GOx hydrogels. (A) Photographs of *S. aureus* colonies (*n* = 3). (B) Plot of live/dead *S. aureus* (scale bar = 100 μm, *n* = 3). (C) SEM images of *S. aureus* (scale bar = 1 μm, *n* = 3). (D) Typical photographs of *S. aureus* biofilms after different treatments (*n* = 3).

After assessing the photothermal heating capability of the hydrogels, the antibacterial effectiveness of the hydrogel was tested by culturing bacteria under 808 nm near‐infrared laser irradiation. We simulated the concentration of H_2_O_2_ in the wound (100 μM) for in vitro antimicrobial testing. We divided into six groups: PBS, H_2_O_2_ + HA/GCA, H_2_O_2_ + HA/GCA + NIR, HA/GCA/Fe^2+^, H_2_O_2_ + HA/GCA/Fe^2+^ and H_2_O_2_ + HA/GCA/Fe^2+^ + NIR. It can be seen through the results that the three groups, PBS, H_2_O_2_ + HA/GCA, and H_2_O_2_ + HA/GCA + NIR, almost did no harm to the bacteria (Figure 4A–C). This suggests that 17.5 μg/mL of GO‐CD warmed to 45°C doesn't have a sterilizing effect. The last group demonstrated the best antibacterial activity, while the HA/GCA/Fe^2+^, H_2_O_2_ + HA/GCA/Fe^2+^, and H_2_O_2_ + HA/GCA/Fe^2+^ + NIR groups gradually reduced the number of bacteria. The experimental results indicate that although 2 mM of Fe^2+^ has some bactericidal effect, the ·OH produced by the Fenton reaction of Fe^2+^ with H_2_O_2_ has a better bactericidal effect. By giving it a slightly hot environment with a 1 W/cm^2^ laser to accelerate the production of ·OH, it can produce a better sterilization effect under the same conditions. Similarly, the same antimicrobial effect was observed for *E. coli* (Figure S9). We also counted the colonies on the smeared plates of *S. aureus* and *E. coli* (Figure S10). Meanwhile, live‐dead staining experiments on different groups of treated bacteria as well as SEM shots for verification yielded results consistent with those of the coated plates.

The treatment of bacterial infectious diseases associated with biofilms is extremely tricky; therefore, we treat the biofilm with different materials. We performed biofilm‐related experiments on both *S. aureus* (Figure 4D) and *E. coli* (Figure S9D). The experimental results show that HA/GCA/Fe^2+^ and H_2_O_2_ + HA/GCA/Fe^2+^ will destroy the biofilm to some extent. Also, H_2_O_2_ + HA/GCA/Fe^2+^ + NIR can destroy most of the biofilm and provide complete elimination of bacteria. Meanwhile, as shown in Figure S10C,D, the lowest OD value was obtained after H_2_O_2_ + HA/GCA/Fe^2+^ + NIR treatment, indicating that this treatment was effective in eliminating the biofilm.

### 2.7 In vivo diabetic wound healing

**FIGURE 5:**
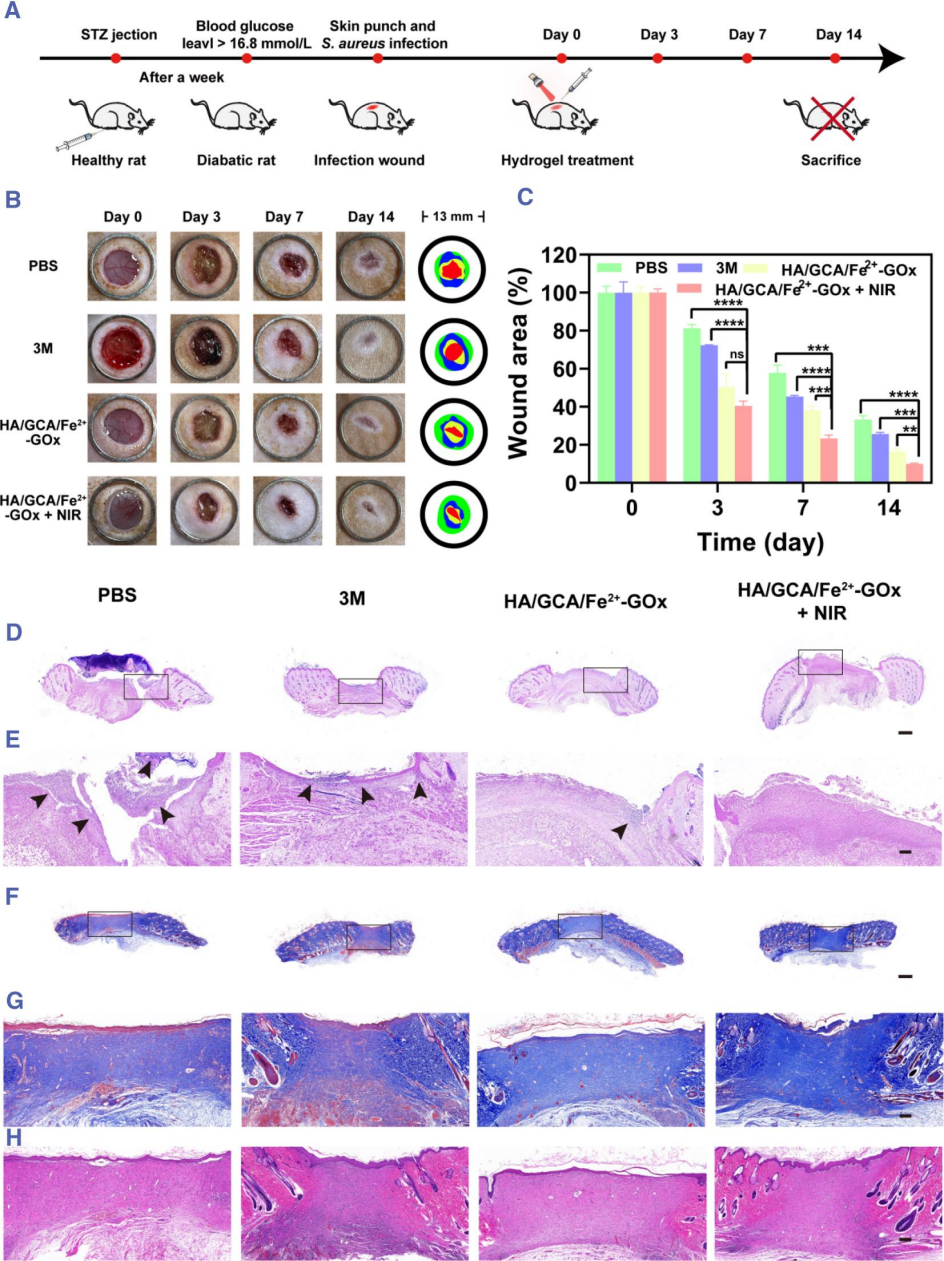
In vitro antimicrobial therapy for diabetic wounds. (A) Create a diagram that illustrates how an infected wound heals. (B) Wound repair in rats under different treatment conditions and superimposed plots of wound repair for different days (*n* = 3). (C) Quantitative evaluation of each group's trauma repair domain (*n* = 3). (D) Gram staining plots of each group on day 3 (scale bar = 1000 μm, *n* = 3). (E) Local magnification of Gram staining, arrows indicate the site of bacterial infection (scale bar = 200 μm, *n* = 3). (F) Masson staining plots for each group on day 14 (scale bar = 1000 μM, *n* = 3). (G) Local magnification of Masson staining map (scale bar = 200 μm, *n* = 3). (H) Localized magnified H&E staining map (scale bar = 200 μm, *n* = 3). (*p < 0.05, **p < 0.01, ***p < 0.001, ****p < 0.0001 and NS means not significant).

By developing a diabetic rat model, we were able to confirm the benefits of HA/GCA/Fe^2+^‐GOx + NIR hydrogel as a wound dressing in vivo and further assess the application of wound healing (Figure 5A). First, the photothermal properties of the gel on rat wounds were investigated. As shown in Figure S11, the skin temperature of the PBS‐treated rats barely increased, whereas the temperature of the HA/GCA/Fe^2+^‐GOx‐treated rats increased from 37°C to 45°C at the wound within 5 min. Following the various treatments, the wounds were collected, and the agar plate method was used to count the bacteria in them (Figure S12). The outcomes were in line with the water antibacterial in vitro findings. The control and 3M groups included the greatest number of germs, but the HA/GCA/Fe^2+^‐GOx groups showed a progressive decline in the number of bacteria. Bacteria were detected in tiny amounts in the HA/GCA/Fe^2+^‐GOx + NIR group.

In a rat hepatic bleeding model, the hemostatic qualities of the HA/GCA/Fe^2+^‐GOx hydrogels were assessed in further detail. Three different treatments were used to measure the amount of blood lost during hemostasis after 5 min of bleeding: gauze, PBS, and HA/GCA/Fe^2+^‐GOx hydrogel. The rats' livers were cut in a few different places. While there was still some bleeding in the other groups, the hydrogel‐treated group exhibited the best hemostasis, as seen in Figure S13, with almost no visible blood present. These findings validated the HA/GCA/Fe^2+^‐GOx hydrogels' strong in vivo hemostatic properties.

Next, we evaluated the in vivo wound healing characteristics of the HA/GCA/Fe^2+^‐GOx hydrogel using a diabetic rat wound model contaminated with bacteria. Two groups were utilized for comparison: HA/GCA/Fe^2+^‐GOx and HA/GCA/Fe^2+^‐GOx + NIR. The PBS group served as the negative control and the 3M gel as the positive control. Photographs were taken at 0, 3, 7, 14 and 15 days after various treatments to document the wound's healing process (Figure 5B). Wounds treated with HA/GCA/Fe^2+^‐GOx and HA/GCA/Fe^2+^‐GOx + NIR healed considerably better than those treated with PBS or 3M. There was minimal change on day 14 between wounds treated with 3M adhesive (25.7%) and those treated with PBS (33.2%), indicating that 3M is ineffective at accelerating the healing of wounds infected with bacteria. Nevertheless, the wound area was decreased to 16.4% and 9.8%, respectively, by the HA/GCA/Fe^2+^‐GOx and HA/GCA/Fe^2+^‐GOx + NIR groups (Figure 5C). It is hypothesized that by eliminating the germs on the wounds, they were able to speed up the healing process; the gel plus light group, in particular, showed the best wound restoration.

In conjunction with the results of wound repair, we used histological staining to study the repair status more visually. We took the skin on day 3 for Gram staining to verify that the wound still had a bacterial infection (Figure 5D–E). It is also evident from the results that the number of bacteria surviving in differently treated wounds varies. The bacteria on the PBS and 3M‐treated groups were almost similar, while there were relatively few bacteria on the HA/GCA/Fe^2+^‐GOx gels. The wounds in the final HA/GCA/Fe^2+^‐GOx + NIR group were almost free of bacteria. This proves that our hydrogel material has good photothermal antimicrobial properties. Meanwhile, we performed H&E staining on the third day for different subgroups of wounds (Figure S14). The results showed significant inflammation in the PBS and 3M groups. In contrast, the HA/GCA/Fe^2+^‐GOx and HA/GCA/Fe^2+^‐GOx + NIR groups had less inflammatory conditions. We took the skin of rats on day 14 of healing for H&E and Masson staining to observe the status of wound repair. From the results, it was also evident that the HA/GCA/Fe^2+^‐GOx + NIR group had the best wound repair with the most collagen deposition (Figure 5F–G), and the H&E sections also showed a small amount of inflammation in the PBS group (Figure 5H). At the same time, we performed collagen statistics on the Masson plot on day 14 (Figure S15). It can be seen that HA/GCA/Fe^2+^‐GOx + NIR‐treated wounds have the most collagen deposits, indicating that wound repair is best after treatment through this group. After that, we took the viscera of rats treated for 14 days, performed H&E staining, and analyzed this. It was found that their hearts, livers, spleens, lungs, and kidneys were virtually free of lesions (Figure S16). It is shown that the gel system is effective in treating bacterial‐infected diabetic wounds while also being biologically safe. From this, we can also demonstrate that our gel plus light can promote the repair of chronic diabetic wounds by sterilizing them.

## 3 CONCLUSION

In summary, this study used ·OH as an initiator to construct an *in situ* injectable HA/GCA/Fe^2+^‐GOx hydrogel for diabetic wound repair. The addition of GOx can consume the glucose of diabetic wounds and provide raw materials (H_2_O_2_) for the Fenton reaction. Furthermore, GO‐CD exhibits excellent photothermal properties, raising the gel temperature to 45°C under 808 nm NIR irradiation. The mild heat (45°C) condition enhanced the Fenton reaction to produce ·OH, with some ·OH used for antibacterial purposes and the rest for gelation, achieving a dual effect. The presence of Ada makes the gel system more compact. Additionally, the HA/GCA/Fe^2+^‐GOx hydrogel demonstrated excellent self‐healing ability and injectability. In vivo experiments have demonstrated that the hydrogel has good biocompatibility and hemostatic and antibacterial effects, promoting the repair of diabetic *S*. *aureus*‐infected wounds. This study provides a promising strategy for *in situ* gel formation and antibacterial agents.

## 4 EXPERIMENTAL METHODS

### 4.1 Materials

Hyaluronic Acid Methacrylated (HAMA, Degree of substitution: 100) was purchased from Wenzhou Shuhe Biotechnology (China) and FeSO_4_ from Shanghai Titan (China). Glucose Oxidase (GOx), methacryloyl chloride, and triethylamine were purchased from Aladdin (China). Ethanol, glutaraldehyde, and MgSO_4_ were purchased from Macklin (China). 1‐Adamantane methanol was purchased from Bidepharm (China). Ethylenediamino‐β‐cyclodextrin (β‐CD) and Graphene oxide (GO) were purchased from Beijing InnoChem (China). Dichloromethane, ethyl acetate, and petroleum aether were purchased from Keshi (China). KOH, NaHCO_3_, HCl, and NaCl were purchased from Huada (China). Streptozotocin (STZ) was supplied by Yuanye (China). Cell Counting Kit (CCK)‐8 was supplied by Beyotime (China). Fetal bovine serum, penicillin‐streptomycin, and Dulbecco's modified Eagle's medium were purchased from Gibco (USA). Solarbio (China) provided the tryptone soya broth (TSB), PBS, and Luria‐Bertani broth (LB). SYTO9 and PI were included in a bacterial live/dead staining kit from Thermo Fisher (L7012, USA).

### 4.2 Synthesis of materials

#### 4.2.1 Synthesis of β‐CD functionalized graphene nanosheets (GO‐CD)

Ethylenediamino‐β‐cyclodextrin (β‐CD) was synthesized following the documented protocol.^23^ GO and β‐CD underwent an epoxide ring‐opening process to create β‐CD functionalized graphene (GO‐CD). A 500 mL round‐bottom flask was usually loaded with 150 mg of GO, and 300 mL of deionized water was added afterward. Following almost 2 h of stirring and ultrasonication, the solution was supplemented with 200 mg of KOH and 1 g of β‐CD. To make sure everything dissolved completely, the liquid was swirled for 10 min at room temperature. After that, it was placed in an oil bath at 80°C and stirred for 24 h. Following the reaction, a uniform black solution was produced. After 30 min of centrifugation at 12,000 g, hardly a little precipitate was seen. The concentration of the solution was done at low pressure. The residue (molecular weight cut off: 1.4104) was put into a dialysis bag. GO‐CD was purified in a basic aqueous solution (pH = 12) for 3 days. The final solution containing GO‐CD inside the dialysis bag was neutralized after 5 days of dialysis with neutral water. The remaining dark solid was dried with vacuum assistance and centrifuged.

#### 4.2.2 Synthesis of adamantanes with grafted double bonds (Ada)

After dissolving in 100 mL of dichloromethane, 5 g (30 mmol) of 1‐adamantane methanol and 7 mL (50 mmol) of triethylamine were chilled in an ice bath. Under nitrogen, dropwise additions of methyl chloride (4.36 mL, 45 mmol) were made to the solution. The reaction was continued at 0°C for a further 30 min and then allowed to room temperature for 24 h. The reaction mixture was progressively rinsed with ice‐cold saturated NaCl, 0.1 M aqueous HCl, and 0.1 M aqueous NaHCO_3_. After being dried over anhydrous MgSO_4_, the organic extracts were filtered and concentrated. The product was refined using silica gel column chromatography with an eluent ratio of 40:1 consisting of petroleum ether and ethyl acetate. After the solvent was removed, the product was recovered as pale crystals with a yield of 68.4%.

### 4.3 Preparation of hydrogel

In summary, 1 mg/mL glucose was first dissolved in HAMA (4%) and placed in one of the test tubes. Then, FeSO_4_ (2 mM), GO‐CD (17.5 μg/mL), Ada (8 μg/mL), and GOx (10 μg/mL) were dissolved in DDW and configured in another test tube. Afterward, the two solutions were mixed in a 1:1 volume ratio, and the mixture was sufficiently mixed to change the solution from a liquid to a gel.

### 4.4 Physicochemical characterization of hydrogel

#### 4.4.1 Characterization of the chemical structure and pore size of hydrogels

After being prepared, the various materials (GO‐CD, HA/Ada, HA/GCA, and HA/GCA/Fe^2+^‐GOx) were refrigerated for 24 h at a temperature of −80°C. Subsequently, the samples were removed and incubated for 24–48 h in a lyophilizer. A Tensor II Fourier Transform Infrared Spectrometer (FTIR, Bruker, Germany) was used to examine the alterations in the chemical groups of the hydrogels. Elemental analyses of the gels were performed using a Feiner benchtop SEM (Phenom Pharos, Phenom, Netherlands). The spectra were recorded over a wavenumber range of 4000^−1^ to 400 cm^−1^. Use the SU8010 field emission scanning electron microscope (FESEM, Hitachi, Japan) to observe the morphological characteristics of the sample. The SEM's acceleration voltage is 5 kV.

#### 4.4.2 Swelling and decongestion test of hydrogels

The developed hydrogels (HA/GC/Fe^2+^‐GOx and HA/GCA/Fe^2+^‐GOx) were lyophilized and submerged in PBS solution for the swelling test. The hydrogels were weighed periodically until their weight stayed consistent after the extra water was removed using filter paper.^24^

(1) $$\text{Swelling}\;\text{ratio}=\frac{W_{\text{real}-\text{time}\;\text{quality}\;\text{of}\;\text{hydrogel}}}{W_{\text{original}\;\text{quality}\;\text{of}\;\text{hydrogel}}}\times 100\%$$

In the deswelling test, the hydrogels that had reached swelling equilibrium were placed in an oven at 37°C. The weight of the hydrogel was recorded at different time intervals until it remained constant. The weight of the hydrogel was recorded at different time intervals until its weight remained constant.^25^

(2) $$\text{Water}\;\text{retention}=\frac{W_{\text{weight}\;\text{of}\;\text{initial}\;\text{swollen}\;\text{weight}}}{W_{\text{de}s\text{wollen}\;\text{weight}\;\text{of}\;\text{the}\;\text{hydrogel}}}\times 100\%$$

#### 4.4.3 Rheological properties of hydrogels

The rheological properties of the produced gels (HA/GC/Fe^2+^‐GOx and HA/GCA/Fe^2+^‐GOx) were assessed using a stress‐controlled DHR‐2 rheometer (TA, USA). The evaluation included the following methods of measurement: (i) Frequency‐scan experiments were conducted at 25°C to investigate the connection between frequency and modulus in the 0.01–100 Hz range, where G′ represents the storage modulus and G″ is the loss modulus. (ii) Shear‐thinning tests were conducted, varying the shear rate from 0.01 to 10 Hz; (iv) Dynamic step‐strain amplitude tests involved alternating the oscillatory strain from 1% to as much as 400% over a brief period; and (v) strain‐scan tests were performed on molded specimens with oscillatory strains ranging from 0.1% to 1000%. (iii) Dynamic oscillatory time‐scan measurements were carried out with 1% strain and a constant frequency of 0.1 Hz.

### 4.5 Photothermal performance

The thermal conversion capacity of the GO‐CD and HA/GCA/Fe^2+^‐GOx gels under near‐infrared (NIR) laser irradiation was examined using photothermal studies. First, 100 μL of GO‐CD (0, 15, 17.5, 20, 25, 50 μg/mL) was added to a 1.5 mL centrifuge tube. Suitable concentrations were screened using 808 nm NIR at 1 W/cm^2^. After determining the concentrations, HA/GCA/Fe^2+^‐GOx gels were prepared (HAMA = 2%, GO‐CD = 17.5 μg/mL, Ada = 8 μg/mL, FeSO_4_ = 2 mM, GOx = 10 μg/mL). The test material underwent irradiation with an 808 nm NIR laser at power levels of 0.5, 1, and 2 W/cm^2^. The samples' temperature was simultaneously recorded using an E4 infrared camera (FLIR, USA). The HA/GCA/Fe^2+^‐GOx gel materials were made, and then they were exposed to NIR irradiation (1 W/cm^2^) until a maximum temperature was reached. This was done to assess the samples' photothermal conversion efficiency. The temperature reaches its peak. A near‐infrared spectrometer with a wavelength of 880 nm was used to record the temperature change and analyze the samples' cyclic photothermal characteristics. Three cycles of 5 min of irradiation time and around 6 min of cooling time were used to examine the samples' cyclic photothermal performance.

### 4.6 Hydroxyl radical detection

Hydroxyl radicals (·OH) were detected using 3,3′,5,5′‐tetramethylbenzidine (TMB). Fe^2+^ in combination with H_2_O_2_ undergoes a Fenton reaction to produce ·OH, whereas TMB is oxidized in the presence of ·OH to produce a blue product. Specifically, different concentrations of FeSO_4_ (20, 60, and 100 μM) were prepared and reacted with the same concentration of H_2_O_2_ (100 μM) with the addition of TMB (10 μM), and the UV‐visible spectra at 650 nm were recorded after the reaction for 5 min at different temperatures (25°C and 45°C).

TA was used as a probe to determine ·OH production. Briefly, hydrogen peroxide (100 μM), TA solution (5 mM) and FeSO_4_ solution (20 μM) were mixed in PBS. After incubation for 10 min at room temperature with an excitation wavelength of 315 nm, the emission wavelength at 435 nm was tested, which is related to the production of ·OH.

### 4.7 In vitro biocompatibility assay

#### 4.7.1 Cytotoxicity detection assay

Various doses of FeSO_4_ (0.5, 1, 2, 4, and 8 mM) were used to create hydrogels. 0.1 g of hydrogel was UV‐sterilized and then incubated for 24 h in 1 mL of cell culture media. Following that, the leachate was protected and the hydrogel was removed. RS1 and L929 cell culture media were injected at a density of 8 × 10^4^ into 96‐well plates. Each well was then filled with 100 μL of gel leachate, and the plates were incubated for 24 h at 37°C with 5% CO_2_. For two to 3 h, the cells were cultured in a fresh medium containing 10% CCK‐8, and their 450 nm OD values were measured.^26^

(3) Cellviability=Treatmentgroup/Controlgroup×100%

#### 4.7.2 Hemocompatibility assessment

First, 1 mL of phosphate buffer PBS was combined with 20 μL of rat blood. Then, 20 mg of hydrogel, PBS, or deionized water is added. Here, PBS is the positive control while deionized water is the negative control. After 4 h at 37°C, the samples were layered and centrifuged for 5 min at 1500 rpm. The resultant supernatant is collected, and a spectrophotometer (1530, Thermo Fisher, Finland) is used to measure it at OD = 540 nm.

(4) $$\text{Haemolysis}\;\text{rate}=\frac{\left({\text{OD}}_{\text{sample}\;\text{group}}\;-\;{\text{OD}}_{\text{negative}\;\text{control}\;\text{group}}\right)}{\left({\text{OD}}_{\text{positive}\;\text{control}\;\text{group}}\;-\;{\text{OD}}_{\text{negative}\;\text{control}\;\text{group}}\right)}\times 100\%$$

### 4.8 In vitro antibacterial assay

#### 4.8.1 Photothermal antibacterial test in vitro

Briefly, the bacterial solution (1 × 10^4^ CFU/mL) was mixed with different materials. We set up six subgroups , including PBS, H_2_O_2_ + HA/GCA, H_2_O_2_ + HA/GCA + NIR, HA/GCA/Fe^2+^, H_2_O_2_ + HA/GCA/Fe^2+^ and H_2_O_2_ + HA/GCA/Fe^2+^ + NIR. The light group was irradiated with an 808 NIR laser for 5 min and then incubated for 1 h. Afterward, the bacterial solution was diluted and 30 μL was spread evenly on an agar plate and incubated overnight at 37°C in an incubator. Bacterial survival in each group was counted for analysis.

#### 4.8.2 Bacteria live/dead staining experiment

The antibacterial capacity of the various groups (PBS, H_2_O_2_ + HA/GCA, H_2_O_2_ + HA/GCA + NIR, HA/GCA/Fe^2+^, H_2_O_2_ + HA/GCA/Fe^2+^, and H_2_O_2_ + HA/GCA/Fe^2+^ + NIR) was examined using the bacterial live/dead staining kit. When bacteria are stained with SYTO9, their undamaged cell membranes show green fluorescence; when bacteria are stained with PI, on the other hand, their cell membrane damage and eventual bacterial death are shown by red fluorescence. After applying several treatments, the bacterial solution was cleansed with PBS, incubated with dyes, and examined using a Leica confocal laser scanning microscope.

#### 4.8.3 Examining the morphology of bacteria

Following various material treatments, *S. aureus* and *E. coli* were collected. Three PBS washes were performed on the bacterial solution. Following that, the bacterial precipitate was extracted by centrifugation. The resultant bacteria were subsequently preserved for an additional night at 4°C in a refrigerator using 2.5% glutaraldehyde. Gradient dehydration with ethanol concentrations ranging from 30% to 90% was the next step in the process. Subsequently, the bacteria were immersed in 99.5% ethanol, and 10 μL was transferred onto a silicon wafer for drying before being examined under an SEM.

#### 4.8.4 Bacterial biofilm formation

*S. aureus* was diluted to 1 × 107 CFU mL^−1^ with TSB medium. Then, 200 μL of bacteria were placed into 96‐well plates and incubated without shaking at 37°C for 36 h. Fresh TSB medium was replaced every 12 h. The bacteria were then incubated in a 96‐well plate with the TSB medium at 37°C for 36 h. The TSB medium was replaced every 12 h with fresh TSB medium. *E. coli* was cultured in the same way using LB medium.^27^

### 4.9 Diabetic wound healing in vivo

#### 4.9.1 Diabetic rat model

Male Sprague‐Dawley (SD) rats (Beijing, China; Spafford Bio) were required to fast for 12 h before receiving an injection of 1% streptozotocin, which killed the rats' pancreatic β‐cells and caused diabetes. These rats showed normal polyphagia, polydipsia, and polyuria habits 1 week later. The rats' fasting blood glucose was assessed three times in a row once these behaviors developed, and the results showed that the diabetes model had been successfully established and the number was higher than 16.7 mmol/L.

#### 4.9.2 Hemostasis test

Firstly, the chest cavity of the rat was dissected and a lobe of the liver was removed and padded on filter paper. Then, a cut was made on the liver to make it bleed. Then, different samples (PBS, gauze, HA/GCA/Fe^2+^‐GOx gel) were covered on the wound and the blood left on the filter paper was observed.

#### 4.9.3 Photothermal antibacterial assay

Firstly, diabetic rats were anaesthetized and perforated (8 mm) on their backs. Then, 20 μL of *S. aureus* solution (1 × 10^8^ CFU/mL) was applied to the rat trauma to establish a bacterial infection model. In the control group, the wounds were treated with PBS; in the experiment group, we divided them into three groups: 3M, HA/GCA/Fe^2+^‐GOx, and HA/GCA/Fe^2+^‐GOx + NIR for comparison. An infrared camera was used to continuously detect and record the temperature of each group of wounds. Following these treatments, the rats were collected the following day and put to death from their back wounds, and an agar plate count was used to count the number of bacteria present.

#### 4.9.4 Diabetic wound repair

Different materials (PBS, 3M, HA/GCA/Fe^2+^‐GOx, and HA/GCA/Fe^2+^‐GOx + NIR) were employed to treat the wounds after a diabetic rat wound infection model was established. The rat back wounds were then photographed and recorded on days 0, 3, 7, and 14 to monitor the healing process.

#### 4.9.5 Histological analysis

Rat wounds were collected on days 0, 3, 7, and 14 in that order. According to histology, we stained the wound with Masson's trichrome, hematoxylin‐eosin (H&E), and Gram on the third day. We stained the wound with hematoxylin‐eosin (H&E) and Masson's trichrome on the 14th day. The analysis of wound healing was done using staining findings.

### 4.10 Statistical analysis

*p* values < 0.05 were considered statistically significant. The data were analyzed using the Student's *t*‐test or a one‐way ANOVA. The statistical data are shown in the graphs as mean ± SD (NS denotes *p* > 0.05, ***p < 0.001, **p < 0.01, *p < 0.05).

## AUTHOR CONTRIBUTIONS

Jianliang Shen, Zhiyong Liao, Xuan Wu, and Chen Zheng conceived and designed the experiments. Xuan Wu and Chen Zheng conducted the material synthesis and the characterizations of the materials. Chen Zheng and Ming Liu performed the catalytic test and photothermal experiments. Jianliang Shen, Xuan Wu, and Chen Zheng contributed to data analysis. Chen Zheng and Xuan Wu wrote the manuscript. Jianliang Shen, Zhiyong Liao, and Xuan Wu supervised the project and revised the manuscript. Each author reviewed the findings, provided feedback on the text, and approved the edited version of this work.

## CONFLICT OF INTEREST STATEMENT

The authors declare that there are no competing interests.

## ETHICS STATEMENT

All animal experiments were conducted following the Wenzhou Medical University Guidelines for the care and use of laboratory animals and approved by the Wenzhou Medical University Animal Ethics Committee with ethical approval number xmsq2023‐1265.

## Supporting information

Figures S1–S16

## Data Availability

The data that support the findings of this study are available from the corresponding author upon reasonable request.

## References

[1] A. K. Dąbrowska , F. Spano , S. Derler , C. Adlhart , N. D. Spencer , R. M. Rossi , Skin Res. Technol. 2018, 24, 165. 29057509 10.1111/srt.12424

[2] a) J. Liu , M. Qu , C. Wang , Y. Xue , H. Huang , Q. Chen , W. Sun , X. Zhou , G. Xu , X. Jiang , Small 2022, 18, 2106172; 10.1002/smll.202106172 35319815

[3] a) S. M. Huang , C. S. Wu , M. H. Chiu , C. H. Wu , Y. T. Chang , G. S. Chen , C. C. E. Lan , J. Dermatol. Sci. 2019, 96, 159; 31761388 10.1016/j.jdermsci.2019.11.004

[4] a) Y. Liang , J. He , B. Guo , ACS Nano 2021, 15, 12687; 34374515 10.1021/acsnano.1c04206

[5] H. Hu , F. Xu , Biomater. Sci. 2020, 8, 2084. 32118241 10.1039/d0bm00055h

[6] a) L. Liu , X. Feng , Y. Pei , J. Wang , J. Ding , L. Chen , Mater. Sci. Eng. C 2018, 82, 25; 10.1016/j.msec.2017.08.045 29025655

[7] Y. Gao , Z. Li , J. Huang , M. Zhao , J. Wu , J. Mater. Chem. B 2020, 8, 8768. 33026387 10.1039/d0tb01074j

[8] a) Y. Gao , S. Meng , W. Liu , Y. Zhang , Y. Zhang , A. Dong , L. Zhang , ACS Appl. Mater. Interfaces 2023, 15, 7735; 36735761 10.1021/acsami.2c19113

[9] S. Li , S. Dong , W. Xu , S. Tu , L. Yan , C. Zhao , J. Ding , X. Chen , Adv. Sci. 2018, 5, 1700527. 10.1002/advs.201700527 PMC5980143 29876202

[10] Z. Yu , X. Li , F. Xu , X. Hu , J. Yan , N. Kwon , G. Chen , T. Tang , X. Dong , Y. Mai , D. Chen , J. Yoon , X. He , H. Tian , Angew. Chem. Int. Ed. 2020, 59, 3658. 10.1002/anie.201913506 31868285

[11] J. Lin , H. Yang , Y. Zhang , F. Zou , H. He , W. Xie , Z. Zou , R. Liu , Q. Xu , J. Zhang , G. Zhong , Y. Li , Z. Tang , Y. Deng , S. Cai , L. Wang , Y. Huang , Y. Zhuo , X. Jiang , W. Zhong , Small 2023, 19, 2205024. 10.1002/smll.202205024 36398604

[12] P. Velander , C. Theopold , T. Hirsch , O. Bleiziffer , B. Zuhaili , M. Fossum , D. Hoeller , R. Gheerardyn , M. Chen , S. Visovatti , H. Svensson , F. Yao , E. Eriksson , Wound Repair Regen. 2008, 16, 288. 18318812 10.1111/j.1524-475X.2008.00367.x

[13] M. Shi , Z. Du , Y. Qi , W. Li , H. Hu , X. Lin , S. Wang , Z. Tang , M. Zhou , Theranostics 2022, 12, 2658. 35401823 10.7150/thno.64244 PMC8965477

[14] Y. Wang , S. Li , H. Yang , J. Luo , RSC Adv. 2020, 10, 15328.35495479

[15] G. Di Mauro , R. Amoriello , N. Lozano , A. Carnasciali , D. Guasti , M. Becucci , G. Cellot , K. Kostarelos , C. Ballerini , L. Ballerini , ACS Nano 2023, 17, 1965. 36692902 10.1021/acsnano.2c06609 PMC9933621

[16] J. Wang , J. Lin , L. Chen , L. Deng , W. Cui , Adv. Mater. 2022, 34, e2108325. 34902192 10.1002/adma.202108325

[17] R. Yu , H. Zhang , B. Guo , Nano‐Micro Lett. 2022, 14, 1. 10.1007/s40820-021-00751-y PMC8639891 34859323

[18] W. Xiu , L. Wan , K. Yang , X. Li , L. Yuwen , H. Dong , Y. Mou , D. Yang , L. Wang , Nat. Commun. 2022, 13, 3875. 35790729 10.1038/s41467-022-31479-x PMC9256606

[19] S. Yougbaré , C. Mutalik , G. Okoro , I. H. Lin , D. I. Krisnawati , A. Jazidie , M. Nuh , C. C. Chang , T. R. Kuo , Int. J. Nanomed. 2021, 16, 5831. 10.2147/IJN.S328767 PMC8405884 34475754

[20] Q. Xu , W. Xiu , Q. Li , Y. Zhang , X. Li , M. Ding , D. Yang , Y. Mou , H. Dong , Mater. Today Bio 2023, 19, 100559. 10.1016/j.mtbio.2023.100559 PMC9926023 36798535

[21] Y. Ding , H. Xu , C. Xu , Z. Tong , S. Zhang , Y. Bai , Y. Chen , Q. Xu , L. Zhou , H. Ding , Z. Sun , S. Yan , Z. Mao , W. Wang , Adv. Sci. 2020, 7, 2001060. 10.1002/advs.202001060 PMC7507500 32995124

[22] W. Ren , Y. Yan , L. Zeng , Z. Shi , A. Gong , P. Schaaf , D. Wang , J. Zhao , B. Zou , H. Yu , G. Chen , E. M. B. Brown , A. Wu , Adv. Healthcare Mater. 2015, 4, 1526. 10.1002/adhm.201500273 26010821

[23] X. Li , Q. Chen , X. Tong , S. Zhang , H. Liu , J. Membr. Sci. 2021, 634, 119350.

[24] D. Humelnicu , M. M. Lazar , M. Ignat , I. A. Dinu , E. S. Dragan , M. V. Dinu , J. Hazard. Mater. 2020, 381, 120980. 31442692 10.1016/j.jhazmat.2019.120980

[25] X. Qi , W. Pan , X. Tong , T. Gao , Y. Xiang , S. You , R. Mao , J. Chi , R. Hu , W. Zhang , H. Deng , J. Shen , Carbohydr. Polym. 2021, 264, 118046. 33910748 10.1016/j.carbpol.2021.118046

[26] K. J. Ornell , D. Lozada , N. V. Phan , J. M. Coburn , J. Mater. Chem. B 2019, 7, 2151. 32073574 10.1039/c8tb03020k

[27] F. Cao , L. Jin , Y. Gao , Y. Ding , H. Wen , Z. Qian , C. Zhang , L. Hong , H. Yang , J. Zhang , Z. Tong , W. Wang , X. Chen , Z. Mao , Nat. Nanotechnol. 2023, 18, 617. 36973397 10.1038/s41565-023-01346-x

