# Supplementary material for: Photothermal‐enhanced in situ supramolecular hydrogel promotes bacteria‐infected wound healing in diabetes
Source: Smart Med. 2024 Feb 23;3(1):e20230047. doi: 10.1002/SMMD.20230047 (PMC11236056; doi:10.1002/SMMD.20230047)
Supplement: Supplementary file 1 — Figures S1–S16 [file SMMD-3-e20230047-s001.pdf]

Supporting Information

Photothermal-enhanced *in Situ* Supramolecular Hydrogel Promotes Bacteria-infected Wound Healing in Diabetes

Chen Zheng[#1], Xuan Wu[2], Ming Liu[3], Yulong Lan[1], Qian Liu[2], Erya Cai[4], Zhiyong Liao[1], Jianliang Shen*[2,3]*

[1]College of Life and Environmental Science, Wenzhou University, Wenzhou, Zhejiang 325035, P. R. China

[2]Zhejiang Engineering Research Center for Tissue Repair Materials, Wenzhou Institute, University of Chinese Academy of Sciences, Wenzhou, Zhejiang 325001, P. R. China

[3]National Engineering Research Center of Ophthalmology and Optometry, Eye Hospital, Wenzhou Medical University, Wenzhou, Zhejiang 325027, P. R. China

[4]School & Hospital of Stomatology, Wenzhou Medical University, Wenzhou, Zhejiang 325027, P. R. China

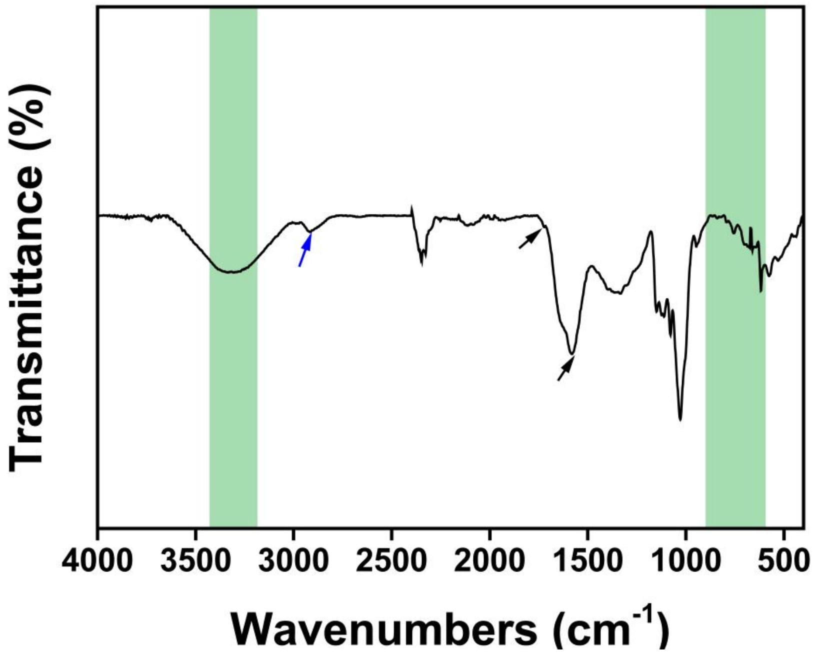

Figure S1. FTIR spectrogram of GO-CD. Where the black arrows indicate the characteristic peaks of GO: the peaks around 1721 cm^{-1} indicate the generation of stretching vibrations of COOH in GO; the peaks around 1578 cm^{-1} indicate the generation of stretching vibrations of COO- in GO. The green boxes indicate the characteristic peaks of β-CD: around 600-900 cm^{-1} indicates the characteristic peaks of bending vibration of the sugar ring; the wider characteristic peaks at 3200-3400 cm^{-1} are the stretching vibration of OH in the sugar ring. The blue arrow around 2850 cm^{-1} indicates the characteristic peak of C-H generated by the covalent bonding of GO with β-CD.

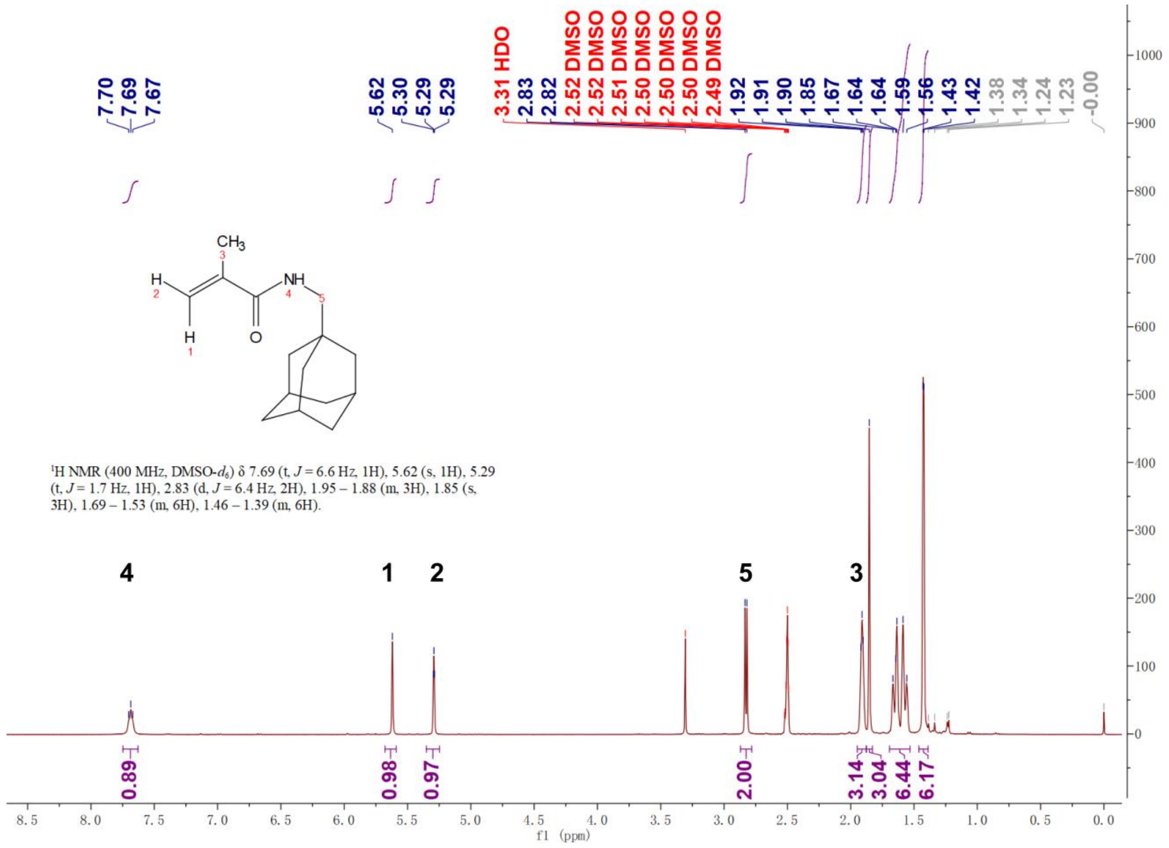

Figure S2. [1]NMR spectrum (400 MHz, DMSO-d6, 298K) of double-bonded adamantane (Ada).

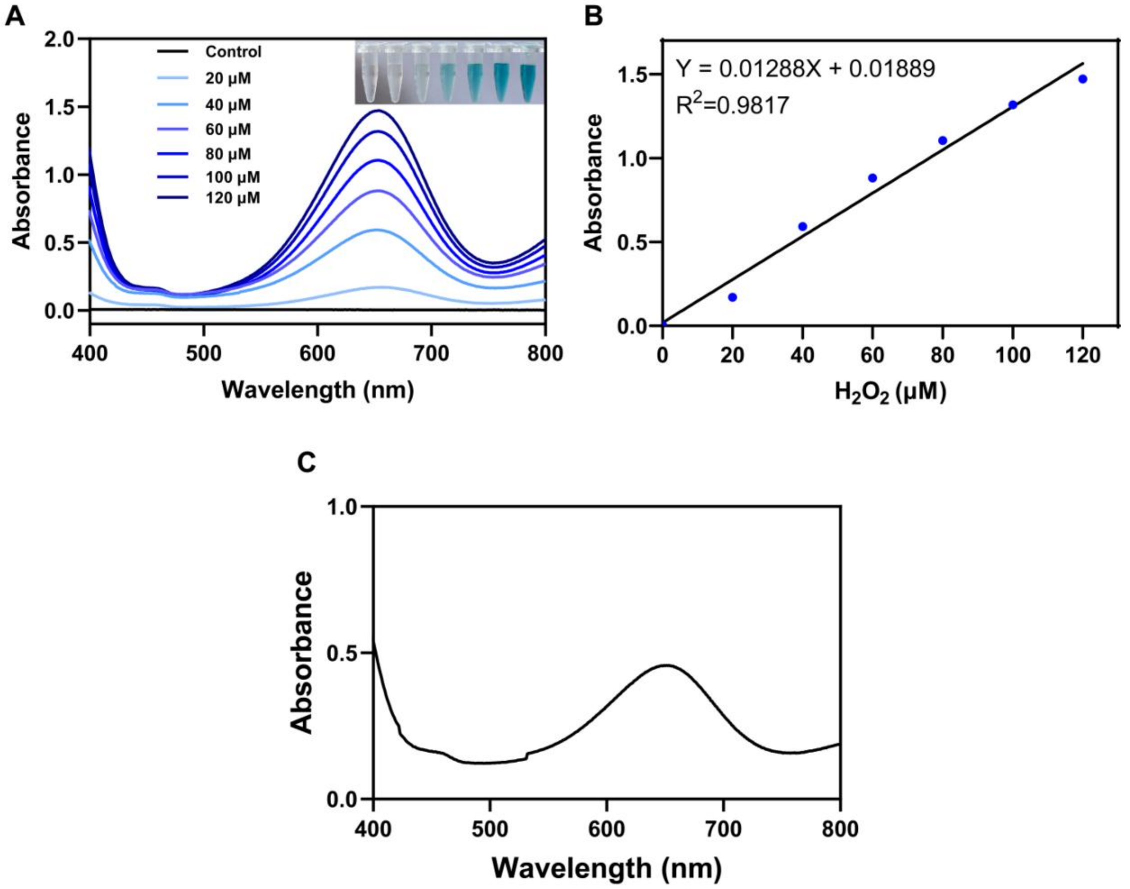

Figure S3. Quantitative detection of H_2O_2 production from the HA/GCA/Fe^{2+}-GOx gel system. (A) The $FeSO_4$ concentration was kept constant (100 μM) and reacted with different concentrations of H_2O_2, the colour change was observed by TMB detection and the UV absorbance was measured. (B) The absorbance value at 650 nm was taken, and linear analysis was done. (C) The concentration of $FeSO_4$ (100 μM, 20-fold dilution over the final sample) was kept constant, GOx = 10 μg/mL, and glucose = 1 mg/mL, and its absorption peak at 650 nm was measured to calculate the approximate amount of H_2O_2 produced by the reaction of the gel system.

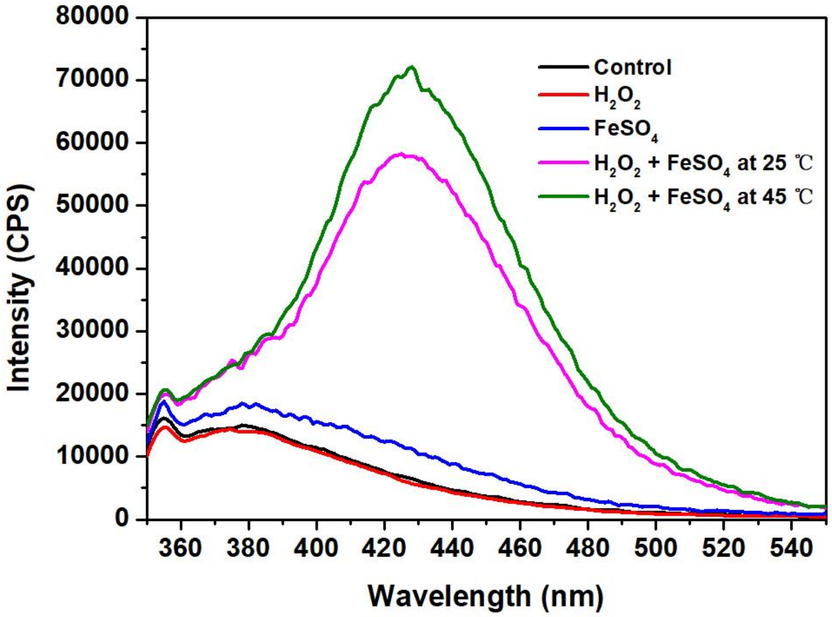

Figure S4. The production of ·OH was determined under different experimental conditions using TA (5 mM) as a fluorescent indicator. where the concentration of H_2O_2 was 100 μM and $FeSO_4$ was 20 μM.

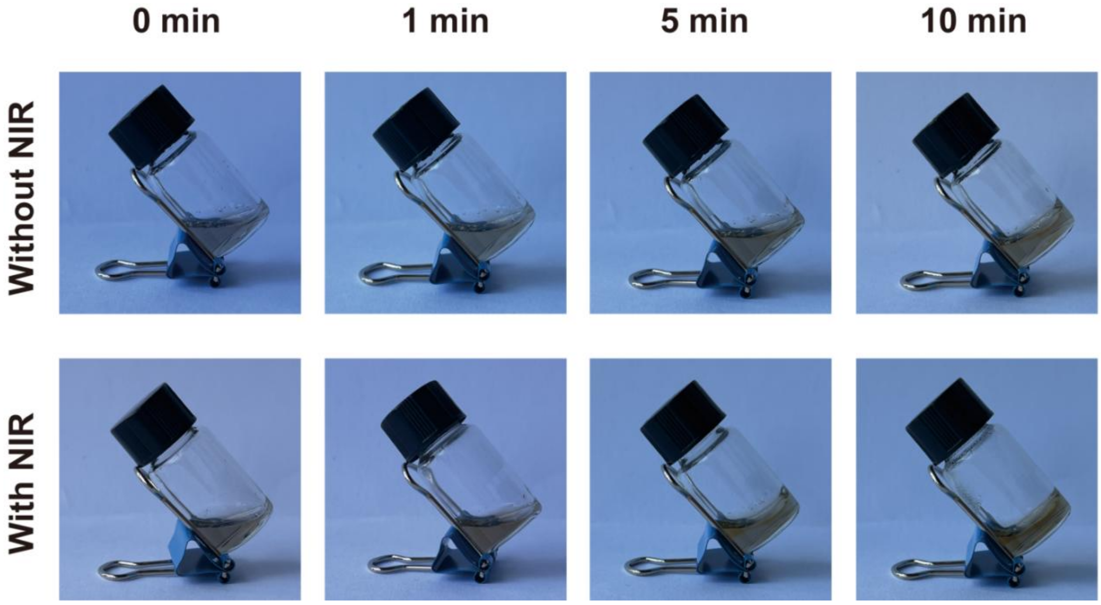

Figure S5. Photothermal promotion of gel formation experiments. $FeSO_4$ (2 mM), HAMA (2%, w/v), GO-CD (17.5 μg/mL), glucose (1 mg/mL), GOx (10 μg/mL), and Ada (8 μg/mL) were divided into light and no light groups to observe the gel formation.

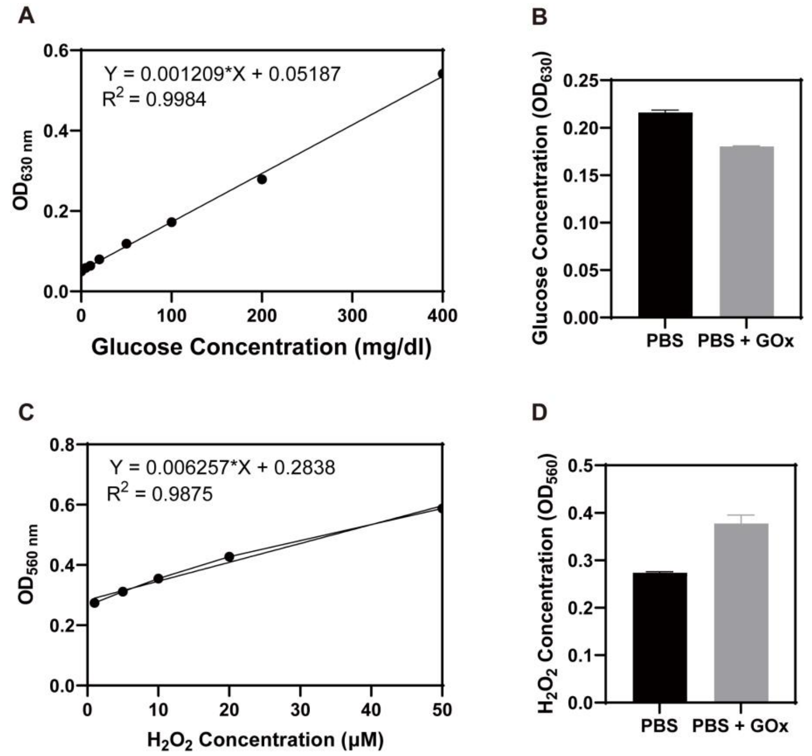

Figure S6. (A) GOx standard curve. (B) Glucose content of the solution before (left) and after (right) GOx treatment. (C)H_2O_2 standard curve. (D) H_2O_2 content of the solution before (left) and after (right) GOx treatment.

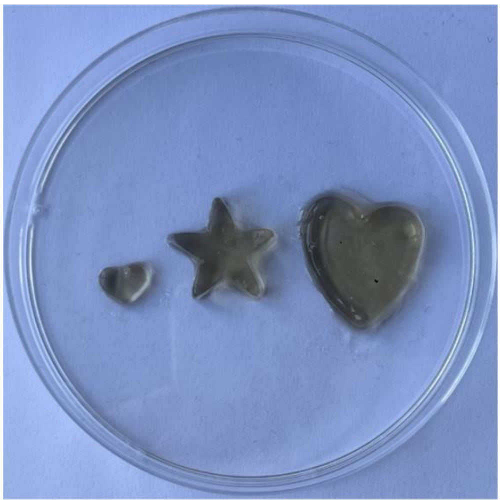

Figure S7. HA/GCA/Fe^{2+}-GOx by in situ formation of gels with different shapes.

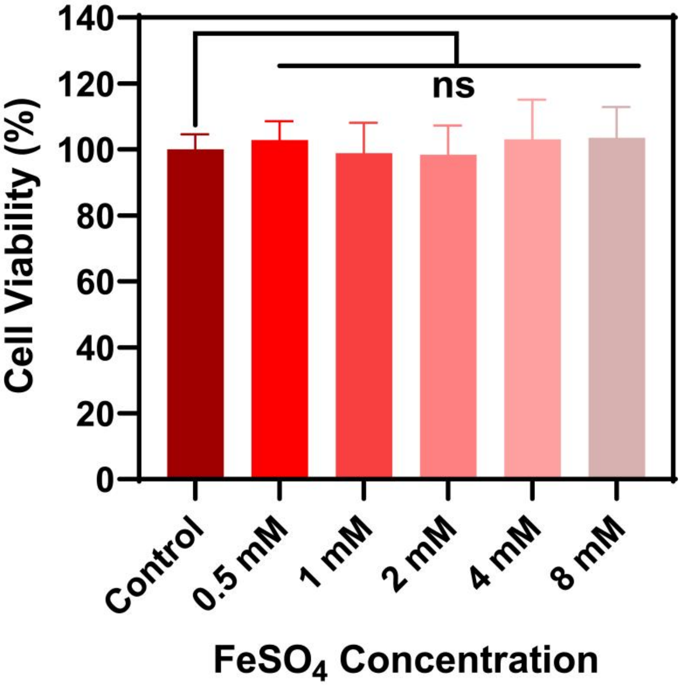

Figure S8. HA/GCA/Fe^{2+}-GOx gels with different $FeSO_4$ concentrations (0.5mM, 1mM, 2mM, 4mM, 8Mm) were prepared, and the gels were immersed in the medium at a volume ratio of 1:100 for 24 h. Afterwards L929 cells were incubated with gel-soaked medium for 24 h for cytotoxicity testing (n = 5). (NS means not significant.).

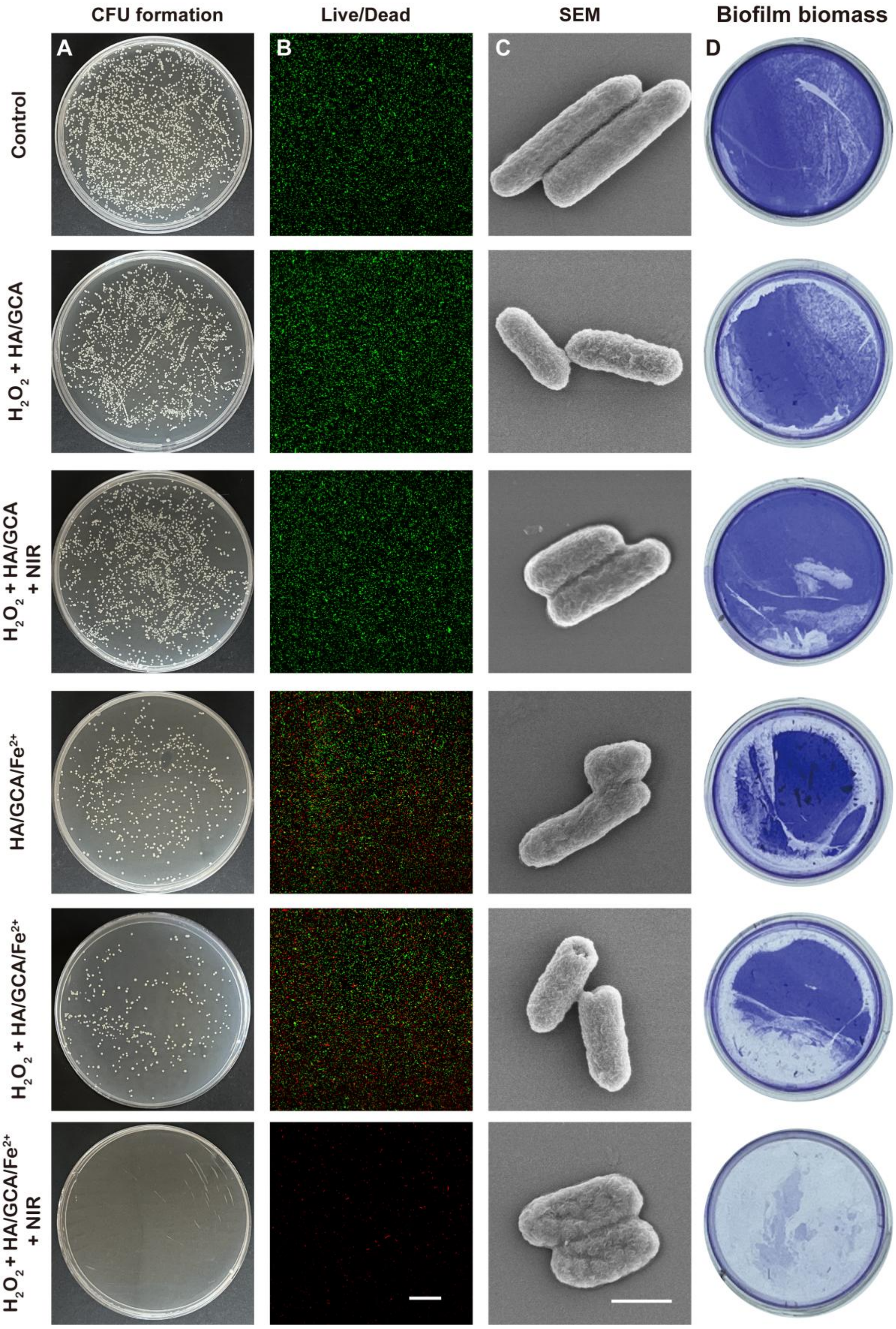

Figure S9. Research on antimicrobial properties of materials. (A) Photographs of *E. coli* colonies (n = 3). (B) Plot of live/dead *E. coli* (scale bar = 100 μm, n = 3). (C) SEM images of *E. coli* (scale bar = 1μm, n = 3). (D) Typical photographs of *E. coli* biofilms after different treatments (n = 3).

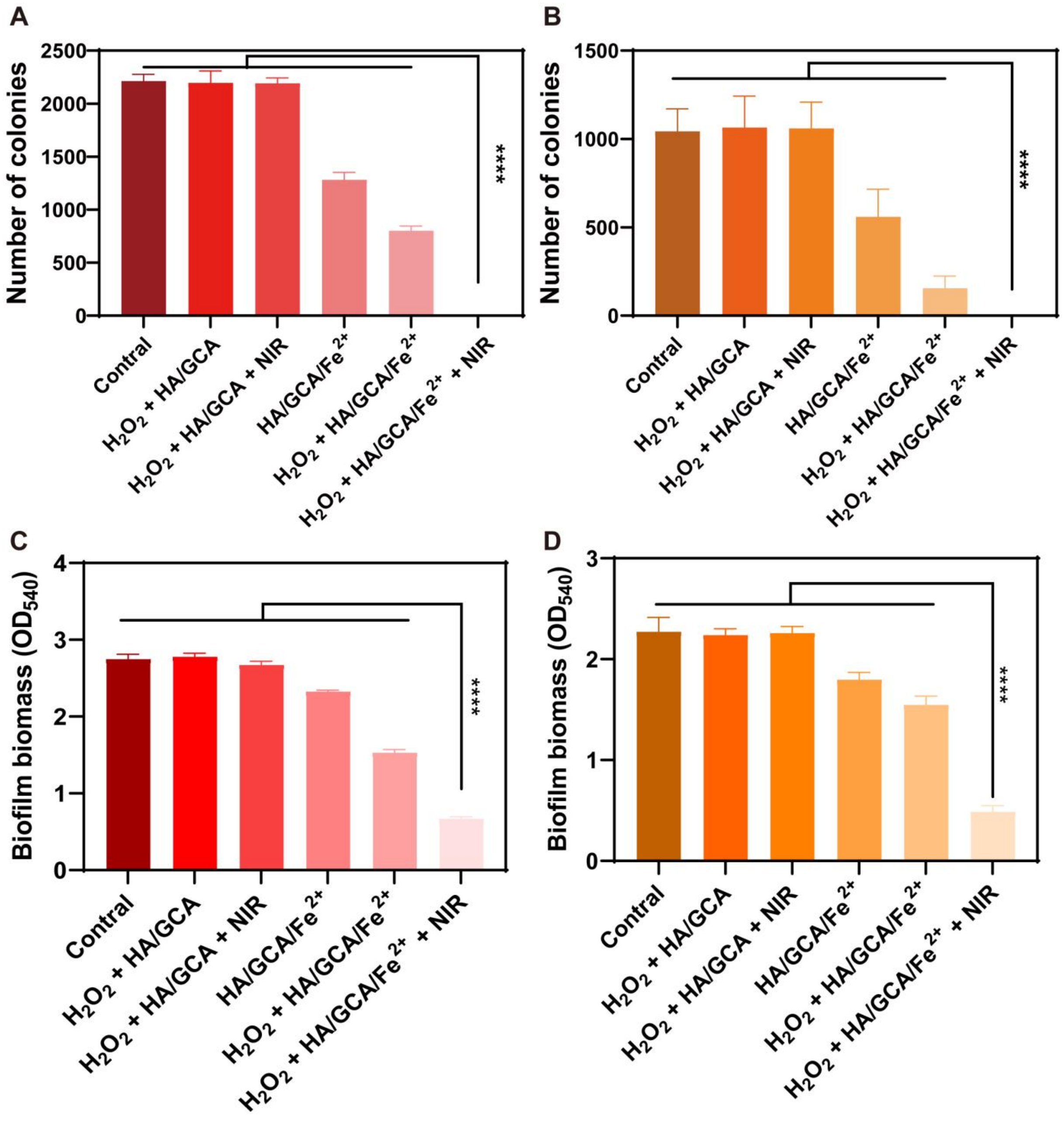

Figure S10. (A) Statistics on the number of colonies of *S. aureus* (n = 3). (B) Statistics on the number of colonies of *E. coli* (n = 3). (C) Corresponding quantitative data for *S. aureus* biofilms (n = 3). (D) Corresponding quantitative data for *E.coli* biofilms (n = 3). (****p < 0.0001).

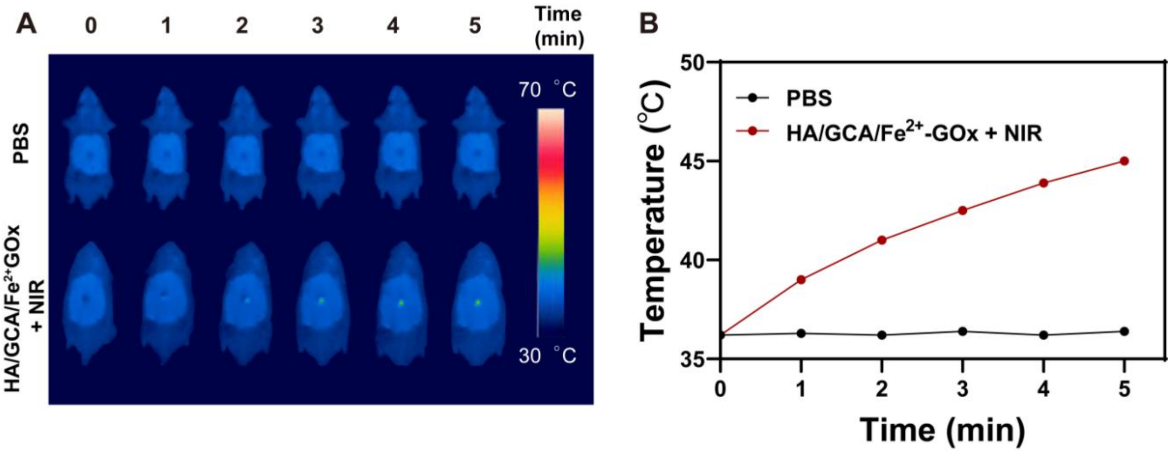

Figure S11. (A) *In vivo* photothermogram of the gel. (B) *In vivo* photothermal warming curves for HA/GCA/Fe^{2+}-GOx gels.

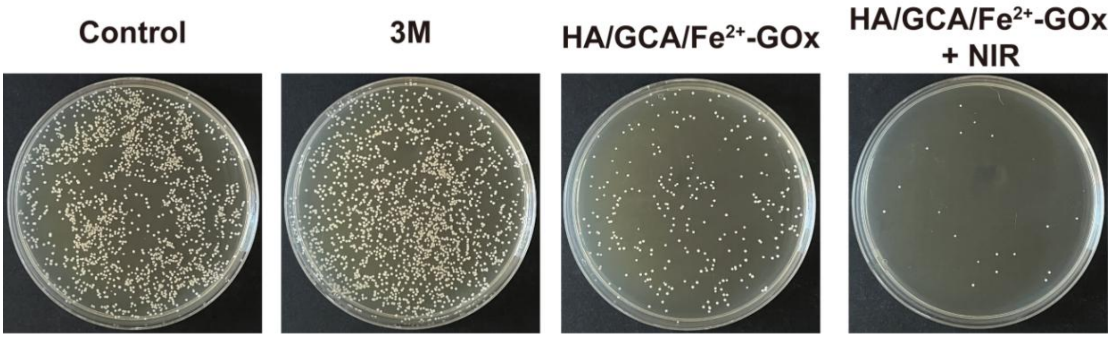

Figure S12. Bacterial plates of wound sites 24 h after treatment in different subgroups.

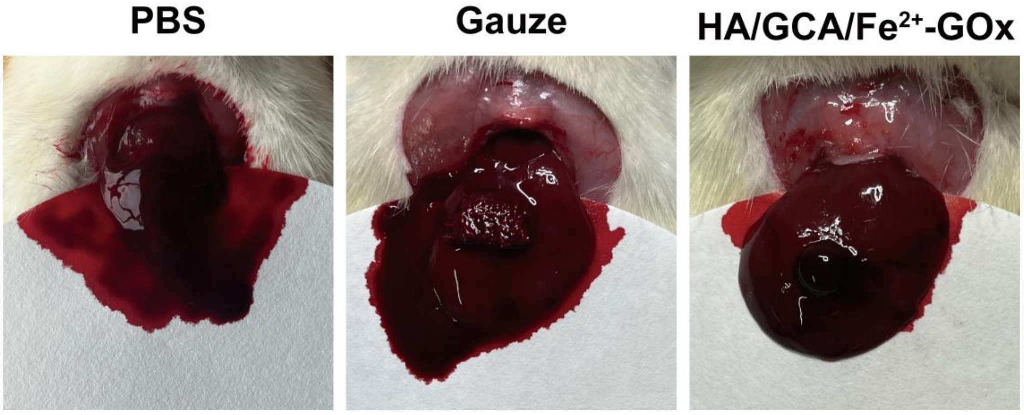

Figure S13. PBS, gauze and HA/GCA/Fe^{2+}-GOx gel hepatic haemostasis test (n = 3).

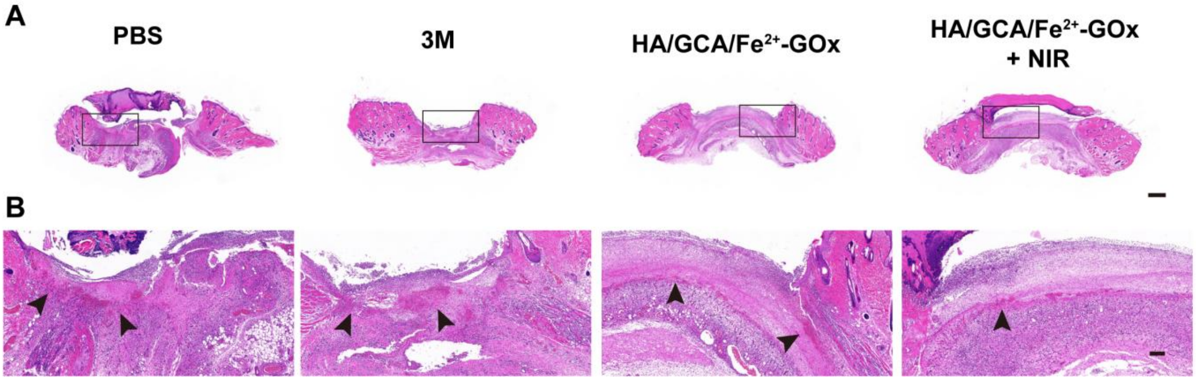

Figure S14. (A) H&E staining on the day 3 (scale bar = 1000 μm, n = 3). (B) Local magnification of H&E staining on day 3, black arrows indicating inflammatory infiltrates (scale bar = 200 μm, n = 3).

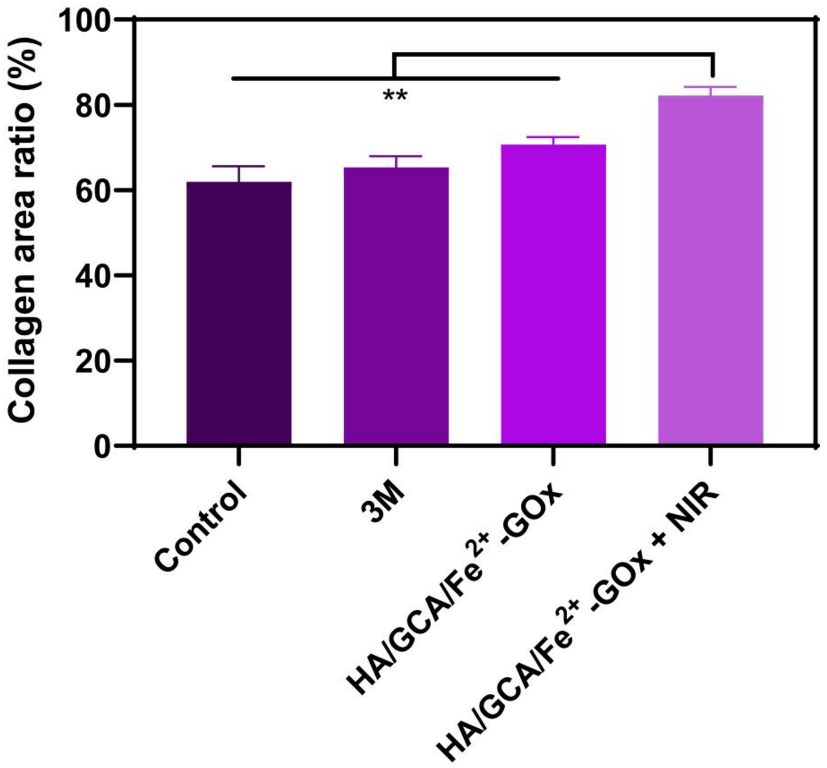

Figure S15. Wound collagen ratio statistics on day 14 of treatment (n = 3). (**p < 0.01).

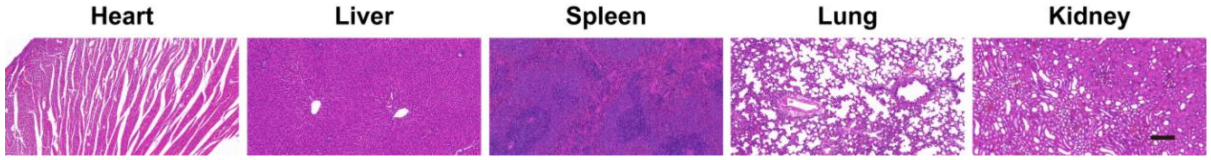

Figure S16. H&E sections of rat viscera from 14 days of treatment (scale bar = 200 μm, n = 3).
